# Leveraging single-cell genomics to expand the fungal tree of life

**DOI:** 10.1038/s41564-018-0261-0

**Published:** 2018-10-08

**Authors:** Steven R. Ahrendt, C. Alisha Quandt, Doina Ciobanu, Alicia Clum, Asaf Salamov, Bill Andreopoulos, Jan-Fang Cheng, Tanja Woyke, Adrian Pelin, Bernard Henrissat, Nicole K. Reynolds, Gerald L. Benny, Matthew E. Smith, Timothy Y. James, Igor V. Grigoriev

**Affiliations:** 1 US Department of Energy Joint Genome Institute, Walnut Creek, CA USA; 2 Department of Plant and Microbial Biology, University of California Berkeley, Berkeley, CA USA; 3 Department of Ecology and Evolutionary Biology, University of Michigan, Ann Arbor, MI USA; 4 Ottawa Hospital Research Institute, Centre for Innovative Cancer Research, Ottawa, Ontario Canada; 5 Architecture et Fonction des Macromolécules Biologiques, UMR 7857 CNRS, Aix-Marseille University, Marseille, France; 6 Institut National de la Recherche Agronomique, USC 1408 Architecture et Fonction des Macromolécules Biologiques, Marseille, France; 7 Department of Biological Sciences, King Abdulaziz University, Jeddah, Saudi Arabia; 8 Department of Plant Pathology, University of Florida, Gainesville, FL USA; 9 Present Address: Department of Ecology and Evolutionary Biology, University of Colorado Boulder, Boulder, CO USA

**Keywords:** Sequence annotation, Fungal genomics

## Abstract

Environmental DNA surveys reveal that most fungal diversity represents uncultured species. We sequenced the genomes of eight uncultured species across the fungal tree of life using a new single-cell genomics pipeline. We show that, despite a large variation in genome and gene space recovery from each single amplified genome (SAG), ≥90% can be recovered by combining multiple SAGs. SAGs provide robust placement for early-diverging lineages and infer a diploid ancestor of fungi. Early-diverging fungi share metabolic deficiencies and show unique gene expansions correlated with parasitism and unculturability. Single-cell genomics holds great promise in exploring fungal diversity, life cycles and metabolic potential.

## Abstract (web summary)

Single-cell genome sequencing of eight uncultured fungal species provides insights into the phylogenetic placement of early-diverging lineages, highlights metabolic deficiencies and identifies gene expansions correlated with parasitism and unculturability.

## Main

Genomics research enables a well-resolved phylogenetic backbone for the fungal tree of life and describes how the fungal nutritional toolkit has evolved over a billion years^1^. A complete grasp of these evolutionary patterns requires a thorough understanding of the enzymes and metabolites utilized by all fungi for accessing diverse sources of nutrition. However, much of what we know about the evolution of the fungal metabolic repertoire is biased towards fungi that are model systems, enzyme factories or important human or plant pathogens^2^. This emphasis results in an underrepresentation of uncultured lineages in the fungal genomic tree and the inaccessibility of these clades to comparative genomic analysis.

Environmental DNA amplicon surveys provide ample evidence for a high diversity of uncultured fungi that are unable to complete their life cycle in axenic conditions. Most studies recover species that are not represented in genomic databases^3–5^. This demonstrates a collectively limited knowledge of the true diversity of fungi, a problem exacerbated when considering early-diverging lineages, which are primarily microscopic and diverse in biotrophic groups^1,6^. Among these uncultured fungal lineages are entire hyperdiverse phyla, such as the Cryptomycota^7^. Even among the culturable early-diverging fungi (EDF), such as the Chytridiomycota, environmental DNA studies show almost no overlap with known and sampled species^8,9^. Because genome sequencing has thus far been limited to cultured fungi, the phylogeny of EDF remains poorly resolved. A better understanding of their genomes will provide clues regarding evolutionary history, basic biology and metabolism (for example, ref. ^10^).

Cultivation-independent methods for sequencing environmental microbial taxa, driven largely by shotgun metagenomics, have been applied for over a decade. Owing in part to the complexity of metagenomic data, the application of single-cell genomics has sharply increased over the past 5 years^11–13^. These methods rely on isolation and lysis of individual cells with subsequent whole-genome amplification and sequencing^14^. Most of the current environmental work has focused primarily on bacterial and archaeal systems^15^ (see ref. ^13^ for a review); however, an increasing number of eukaryotic genomes have been reported from single-cell sequencing^16–19^. Although recent efforts successfully demonstrated both single-nucleus de novo genome sequencing of the endomycorrhizal fungus *Rhizophagus irregularis*^20^ and high-throughput microfluidics single-cell sequencing^21^, these methods are neither generalizable nor easily adoptable in other laboratories. Fungi pose several challenges for scaling up single-cell genomics. These include structural challenges, such as the presence of a cell wall and diverse cellular morphologies, as well as genomic challenges, such as multiple chromosomes and higher ploidy, mitochondrial genomes, wide GC variation and transposable elements. To develop methods for robust capture and de novo assembly of environmental fungal genomes, we designed a project using known target species to explore the technical challenges prior to isolating and sequencing more complex environmental samples.

Here, we applied single-cell genomics methods for the first time to uncultured mycoparasitic EDF from the Cryptomycota, Chytridiomycota and Zoopagomycota^22^: *Rozella allomycis*, *Caulochytrium protostelioides*, *Dimargaris cristalligena*, *Piptocephalis cylindrospora*, *Syncephalis pseudoplumigaleata* and *Thamnocephalis sphaerospora*. We focused primarily on biotrophic mycoparasites because they are an understudied group of fungi that are widely represented among EDF^23^ and they sporulate abundantly when cultured with their hosts. We also included the pollen saprotroph *Blyttiomyces helicus* (Chytridiomycota) and *Daphnia* parasite *Metschnikowia bicuspidata* (Ascomycota) because samples of these fungi also contain DNA from numerous non-target species, similar to environmental samples. In this study, we provide genomic insights into the biology and evolutionary histories of these uncultivated species. We illustrated robust placement of novel lineages among EDF, demonstrated higher than haploid ploidy as a common characteristic of these lineages and revealed interesting gene family evolution patterns outside the Dikarya. We also highlighted common metabolic deficiencies among uncultured lineages and tested whether these deficiencies could be overcome through culturing efforts with addition of limiting reagents. Collectively, these approaches will facilitate further study on diverse and uncultured environmental eukaryotes.

## Results

### Single-cell pipeline successfully captures fungal genomes with high completeness

We developed and applied our single-cell pipeline (Supplementary Fig. 1) to eight diverse target species. We recovered genomes from individual cells (‘1-cell’), as well as pools of multiple cells sequenced as one library (‘10-/30-/50-/100-cells’). These individual libraries (Supplementary Table 1) were combined in separate co-assemblies (a technique routinely used in microbial single-cell projects^15,24,25^) to maximize completeness.

**Fig. 1:**
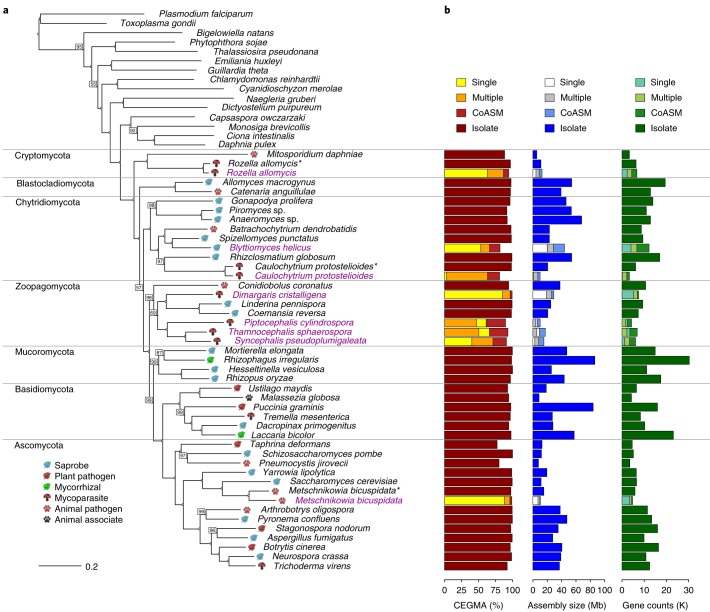
Phylogenetic tree with assembly and annotation statistics. **a**, RAxML tree constructed from MCL clustering from across the fungi and deep-branching eukaryote outgroups. Support values are based on 1,000 bootstrap replicates. Bootstrap values (<100%) are indicated on branches. Fungal species are annotated with simplified lifestyle icons. Species sequenced using single-cell methods are coloured in magenta. Enrichment cultures for *R.* *allomycis* and *C.* *protostelioides* and a related species of *M.* *bicuspidata* are denoted with an asterisk. **b**, Estimated completeness (CEGMA (%)), assembly size and gene model statistics for fungal genomes. Metrics for the most complete individual libraries are indicated on the respective graphs, either as ‘single’ (referring to 1-cell libraries), ‘multiple’ (referring to 100-cell libraries in all cases except for *T.* *sphaerospora*, in which it refers to a 50-cell library) or ‘CoASM’ (referring to co-assemblies). ‘Isolate’ refers to assemblies derived from genomic material sequenced using non-single-cell methods.

**Fig. 2:**
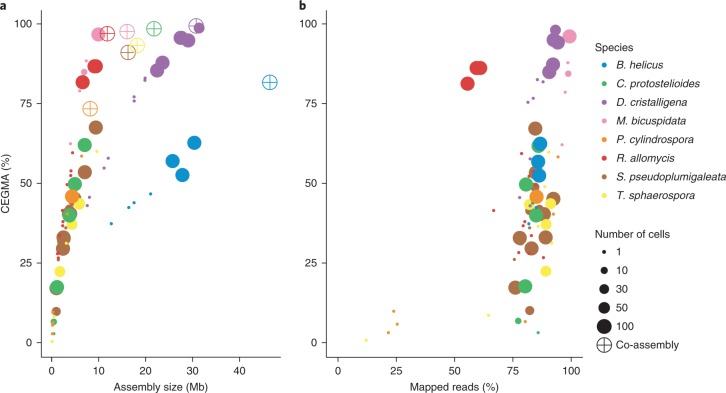
CEGMA correlation with assembly size and read mapping. **a**, Scatter plot showing the correlation between estimated completeness and assembly size for individual libraries and co-assemblies for a given species. **b**, Scatter plot showing the correlation between estimated completeness and the percentage of reads from a given library mapped to the co-assembly for that organism. Individual library sizes are reflected in the size of the points.

Figure 1b summarizes genome completeness (per cent CEGMA (Core Eukaryotic Genes Mapping Approach)^26^), assembly size and total gene content of co-assemblies and individual assemblies. Generally, CEGMA percentages increased when more cells were incorporated into a library, ranging from 2.8% (1 cell, *C.* *protostelioides*) to 96.7% (100 cell, *M.* *bicuspidata*). For all species, co-assemblies were most complete (relative to the 1-cell or 50-cell/100-cell libraries), ranging from 73.36% (*P.* *cylindrospora*) to 99.34% (*D.* *cristalligena*). Assembly sizes of the 1-cell libraries range from 0.5 Mb (*C.* *protostelioides*) to 21.1 Mb (*B.* *helicus*). The largest single-library assembly was 30.1 Mb (100 cell, *B.* *helicus*). Co-assembly sizes ranged from 10.6 Mb (*C.* *protostelioides*) to 46.5 Mb (*B.* *helicus*). Predicted gene counts ranged from 111 (1 cell, *C.* *protostelioides*) to 6,941 (100 cell, *D.* *cristalligena*) for single-library assemblies. Co-assembly gene counts ranged from 3,328 (*C.* *protostelioides*) to 12,167 (*B.* *helicus*). Overall, there is a strong positive correlation between estimated genome completeness and assembly size (Fig. 2a). Within a single taxon, the trend suggests that more cells sorted per library results in more complete libraries. With the exception of *R.* *allomycis*, >75% of reads of a given library were incorporated into an assembly (Fig. 2b).

### Single-cell assemblies provide robust fungal phylogeny

To help resolve fungal phylogeny, a maximum likelihood tree was built from whole-genome clustering data from the eight co-assemblies along with major fungal and deep-branching eukaryotic representatives (Supplementary Table 2). The phylogenetic placement of our co-assembled genomes agrees with current taxonomic understanding: *D.* *cristalligena* groups with Kickxellomycotina; *B.* *helicus* and *C.* *protostelioides* with Chytridiomycota; *R.* *allomycis* with Microsporidia as sister to the rest of the fungi; and *P.* *cylindrospora*, *S.* *pseudoplumigaleata* and *T.* *sphaerospora* form a monophyletic group sister to the Kickxellomycotina within the Zoopagomycota (Fig. 1a).

Because single-cell libraries will only recover a partial genome, we determined the effect of genome completeness and input cell number on generating an accurate phylogeny. We annotated all libraries from *D.* *cristalligena*, *C.* *protostelioides*, *R.* *allomycis* and *M.* *bicuspidata* and constructed individual phylogenetic trees using each library (Supplementary Data). These topologies were compared to the co-assembly-based tree in Fig. 1a. Only three libraries (7.7%) were incongruent for that particular taxon, despite libraries having as few as 15 genes belonging to orthologous gene clusters (median: 434): one *R.* *allomycis* (1 cell, 1,095 clusters) and two *C.* *protostelioides* (100 cell and 10 cell, 1,091 and 1,090 clusters, respectively) (Supplementary Table 3). These results show that less-complete single-celled libraries can be used to generate accurate phylogenies.

### Single-cell genomes indicate that higher ploidy is common in EDF

**Fig. 3:**
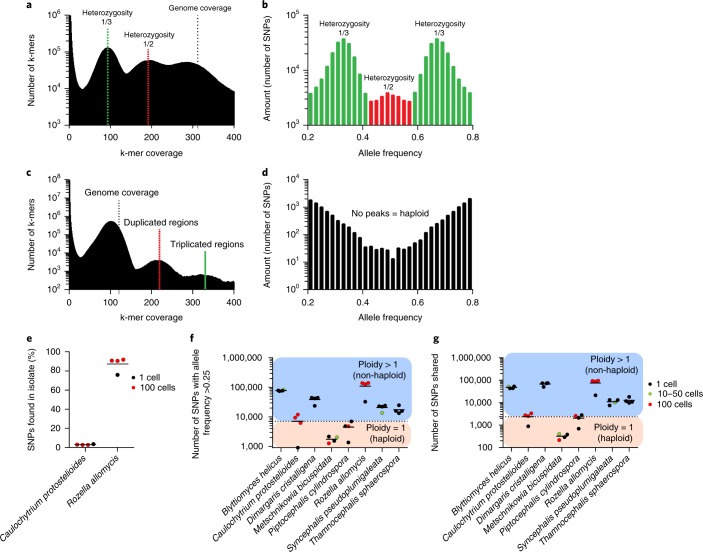
Ploidy analysis. **a**–**g**, Analysis of diversity in population sequencing (**a**–**d**) and single-cell sequencing (**e**–**g**). *R.* *allomycis* (**a**,**b**) and *C.* *protostelioides* (**c**,**d**) population variability is shown in the k-mer distribution plot (**a**,**c**) and in the allele frequency spectra (**b**,**d**). In panel **e**, recovery of isolate SNPs from a single cell (1) and multiple cells (1–100) are shown. In panel **f**, the amount of heterozygous SNPs (allele frequencies above 25%) in single-cell and multiple-cell libraries is indicative of higher ploidy. In panel **g**, high numbers of shared heterozygous SNPs in non-haploid organisms are shown. For **e**–**g**, *n* = 4 biologically independent samples, in which each dot corresponds to one library. The horizontal black lines indicate the mean values.

Single-cell genomes provide a unique opportunity to separate polymorphisms among cells from polymorphisms within cells. The ploidy of most EDF species is largely unknown but a few have been shown to be higher than haploid^27–29^. Analyses with k-mer graphs and allele frequency spectra can successfully distinguish between haploid and non-haploid organisms^29–31^. For *C.* *protostelioides* and *R.* *allomycis*, our results indicate haploid and triploid patterns, respectively (Fig. 3a–d). We were able to identify putative single-nucleotide polymorphisms (SNPs) in single-cell libraries of both species, but only for *R.* *allomycis* do single-cell SNPs match those identified in isolate sequencing (Fig. 3e).

Single-cell sequencing can also identify heterozygous SNPs in diploid cells^32^. By using *C.* *protostelioides* and *R.* *allomycis* as haploid and non-haploid models, respectively, we identified five higher-than-haploid species (Fig. 3f). Moreover, because we are able to show that most variants identified in our five non-haploid species are present in multiple libraries (Fig. 3g), our patterns are consistent with heterozygous SNPs expected in all cells of an isolate. These results show that single-cell sequencing can elucidate the ploidy of fungi and suggest that the majority of EDF are non-haploid.

We also identified cell-to-cell polymorphisms among one-cell libraries of *D.* *cristalligena* (Supplementary Fig. 2). We determined that six SNPs for a particular gene were exactly shared between two libraries (AHPZW and AHPZP), whereas one was unique to a third library (AHSAA).

### Single-cell genomes highlight common deficiencies in primary metabolism of uncultured fungi

Genome sequencing of uncultured EDF allows us to explore how metabolic content has changed during the course of fungal evolution. Compared to the common ancestor of fungi (Supplementary Fig. 3), *D.* *cristalligena* shows gains in high-level metabolic categories, whereas *B.* *helicus* has mainly losses.

**Fig. 4:**
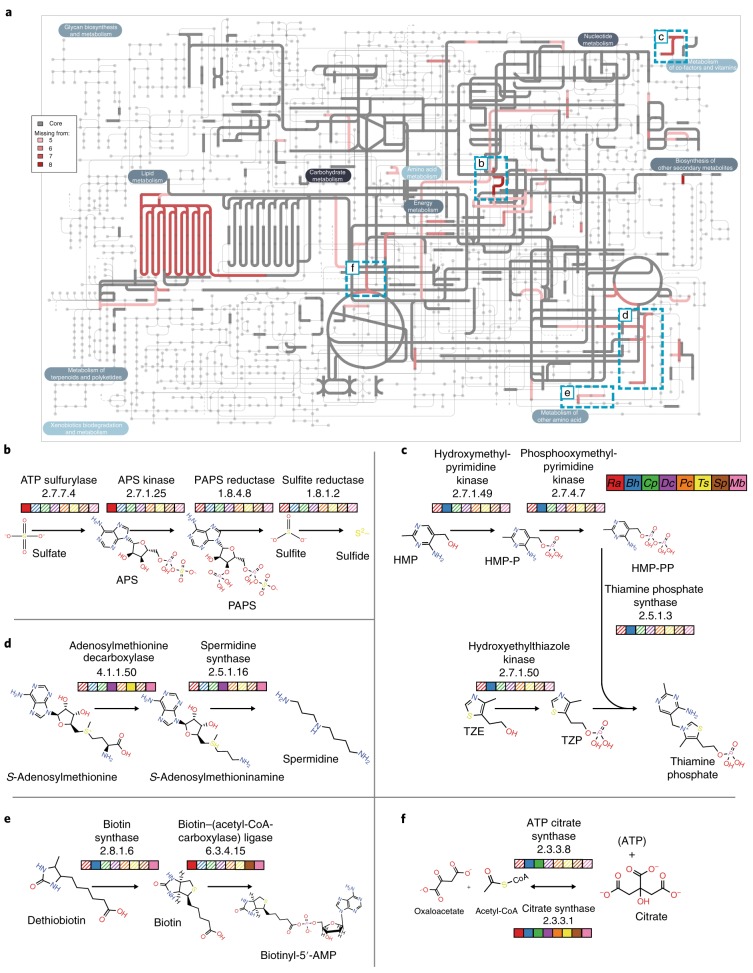
Core metabolism and individual pathways. **a**, Metabolic pathway map with enzymes found in ~70% of ‘free-living’ fungi and absent in at least one of the single-cell fungi shown as a gradient: darker red shades indicate more single-cell species missing the same enzyme in a given pathway. **b**–**f**, Certain pathways with a high degree of such common losses: assimilatory sulfate reduction (sulfate to sulfide) (**b**), thiamine biosynthesis (HMP and TZE to thiamine phosphate) (**c**), spermidine synthesis (*S*-adenosylmethionine to spermidine) (**d**), biotin metabolism (dethiobiotin to biotin (and biotinyl-5′-AMP)) (**e**), and citrate synthesis (citrate to/from oxaloacetate and acetyl-CoA) (**f**). APS, adenylyl sulfate; HMP, 4-amino-5-hydroxymethyl-2-methylpyrimidine; HMP-P, HMP phosphate; HMP-PP, HMP diphosphate; PAPS, phosphoadenylyl sulfate; TZE, 5-(2-hydroxyethyl)-4-methylthiazole; TZP, TZE phosphate. The coloured boxes for **b**–**f** indicate the presence (solid) or the absence (shaded) of a given enzyme in a genome: *Ra*, *R. allomyci*s (red); *Bh*, *B.* *helicus* (blue); *Cp*, *C.* *protostelioides* (green); *Dc*, *D.* *cristalligena* (purple); *Pc*, *P.* *cylindrospora* (orange); *Ts*, *T.* *sphaerospora* (yellow); *Sp*, *S. pseudoplumigaleata* (brown); *Mb*, *M. bicuspidata* (pink).

Examination of the primary metabolism of genomes can reveal deficiencies responsible for unculturability, ideally resulting in successful culturing through supplementing media with missing metabolites^33^. One challenge with predicting missing function using single-cell genomes is the presence of false negatives from missing data, which we address by searching for commonalities in missing pathways across our target taxa. The conserved pathways found in 75% of ‘free-living’ fungi, overlaid with those missing from ≥5 target fungi, reflect consistent enzymatic losses (Fig. 4a).

The absence of spermidine synthase (enzyme classification (EC) 2.5.1.16) and *S*-adenosylmethionine decarboxylase (EC 4.1.1.50) suggests that almost all target taxa are unable to make spermidine and/or homospermidine (Fig. 4d). Spermidine is involved in the regulation of processes such as virulence and sporulation^34,35^, and deficiency in polyamines leads to auxotrophy and attenuation of virulence^36,37^.

Another deficiency common to almost all of the target genomes is in the assimilatory sulfate reduction pathway^38^ (Fig. 4b). ATP sulfurylase (EC 2.7.7.4) and adenylyl-sulfate kinase (EC 2.7.1.25) are missing from all except *R.* *allomycis* and a culturable *M.* *bicuspidata* relative (hereafter: NRRL YB-4993). Phosphoadenylyl-sulfate reductase (EC 1.8.4.8) and sulfite reductase (NADPH) (EC 1.8.1.2) are missing from all target genomes except *M.* *bicuspidata* NRRL YB-4993.

Metabolism of biotin is another common deficiency among target genomes (Fig. 4e). In plants, it is synthesized from dethiobiotin by biotin synthase (EC 2.8.1.6)^39^. This enzyme is only found in *M.* *bicuspidata* and *B.* *helicus*. Similarly, biotin–(acetyl-CoA-carboxylase) ligase (EC 6.3.4.15) is only found in *M.* *bicuspidata*, *R.* *allomycis* and *S.* *pseudoplumigaleata*.

Biosynthesis of thiamine phosphate is accomplished via thiamine phosphate synthase (EC 2.5.1.3) and hydroxyethylthiazole kinase (EC 2.7.1.50). These enzymes are absent from all target fungi except *B.* *helicus*. Both enzymes are absent from *M.* *bicuspidata* from *Daphnia* but are found in *M.* *bicuspidata* NRRL YB-4993 (Fig. 4c).

Entrance into the tricarboxylic acid cycle is accomplished through citrate synthase (EC 2.3.3.1), which is found in all free-living and target fungi. This enzyme facilitates the conversion of acetyl-CoA to citrate. ATP citrate synthase (EC 2.3.3.8) facilitates the same reaction but also generates ATP in the process. It is absent from all target fungi except for the chytrids *C.* *protostelioides* and *B.* *helicus* (Fig. 4f).

Based on our primary metabolism results, we attempted preliminary media-supplementing axenic culturing experiments for three mycoparasitic taxa: *D.* *cristalligena*, *S.* *pseudoplumigaleata* and *P.* *cylindrospora*. We tested the efficacy of five supplements to produce axenic growth of these fungi and obtained mixed results. *S.* *pseudoplumigaleata* either did not grow or was contaminated by the host fungus. *P.* *cylindrospora* grew axenically but weakly on all media treatments, including the control plates, but was not able to complete its life cycle on any of the media formulations. *D.* *cristalligena* responded best to our experiments; this fungus grew faster and more abundantly on the media that included all five supplements when compared to all other treatments. However, even on the fully supplemented axenic media, *D.* *cristalligena* was not able to complete its life cycle (Supplementary Fig. 4). Further experiments will be necessary to fully characterize axenic growth of these and related mycoparasitic EDF (see the Supplementary Information for complete experimental details).

### Proteinase and CAZymes reveal ecology of uncultured fungi

**Fig. 5:**
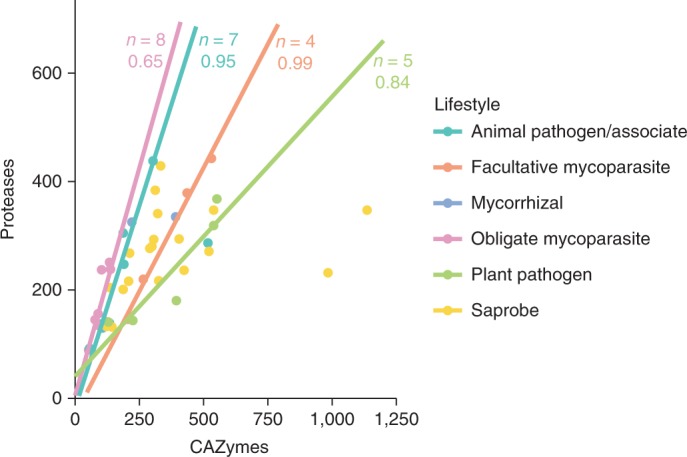
CAZY-to-protease ratios. CAZymes and proteases (defined as in the CAZY database and the MEROPS database) were predicted for each species in the taxon list. Total counts of each were plotted to describe the overall ratio. Correlation was determined using the linear model function (lm) in R, which implements QR decomposition. A trend line for saprobe species (*n* = 19) was omitted owing to a low *r*^2^ value. Similarly, a trend line for mycorrhizal species was omitted owing to a low species count (*n* = 2).

To explore parasitism strategies, we focused broadly on abundance patterns of enzymatic protein domains. Comparing carbohydrate-active enzymes (CAZymes; http://www.cazy.org)^40^ and proteases (MEROPS; https://www.ebi.ac.uk/merops/)^41^, we found that facultative mycoparasites have a CAZyme-to-peptidase ratio most similar to plant pathogens, whereas that for obligate mycoparasites is most similar to animal pathogens (Fig. 5). *B.* *helicus*, the only saprotroph in this data set, has the most CAZymes among our single-cell genomes, with expansions of families that target pollen polysaccharides (Supplementary Table 4). To complement these broad observations, we focused narrowly on families often associated with mycoparasitism: subtilases, metallopeptidases and chitinases.

### Subtilases in Zoopagomycota

**Fig. 6:**
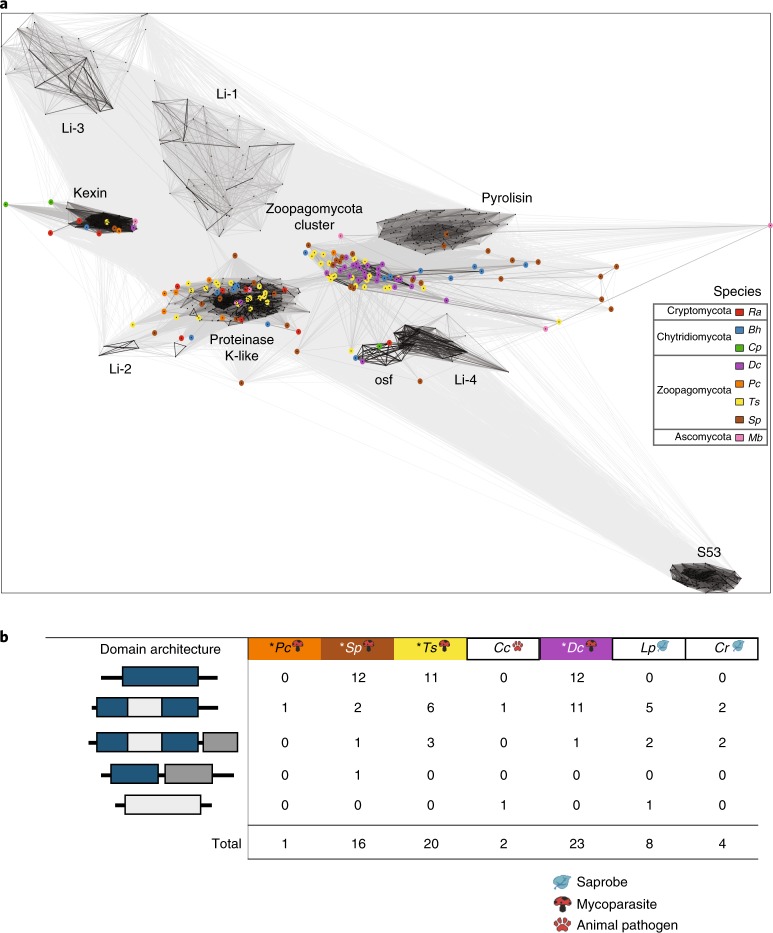
Subtilases. **a**, Network figure highlighting the relationships between fungal subtilases. The distance reflects sequence similarity. Groups marked as Li-1 through to Li-4 refer to clusters previously reported by Li et al.^45^, from which the data set originated. *Bh*, *B.* *helicus*; *Cp*, *C.* *protostelioides*; *Dc*, *D.* *cristalligena*; *Mb*, *M.* *bicuspidata*; *Pc*, *P.* *cylindrospora*; *Ra*, *R.* *allomycis*; *Sp*, *S.* *pseudoplumigaleata*; *Ts*, *T.* *sphaerospora*. **b**, Subtilase domain architecture in Zoopagomycota genomes, for proteins in a given homologous cluster. Mycoparasites sequenced using single-cell methods from this study (labelled with *) have generally more subtilase proteins. For *S.* *pseudoplumigaleata* and *T.* *sphaerospora*, these are predominately single-domain (PF00082) proteins. Domain architecture colours: blue, PF00082; light grey, PF02225; dark grey, PF06280. Lifestyles are indicated with simplified icons. Species abbreviations are consistent with panel **a** and also include: *Cc*, *Conidiobolus coronatus*; *Cr*, *C.* *reversa*; *Lp*, *L.* *pennispora.*

Subtilases, one of the largest clans of serine endopeptidases, are found in fungal entomopathogens, mycoparasites and plant pathogens^42,43^. Recent work has characterized novel categories of serine proteases in fungi^44,45^ in the Dikarya. We explored subtilase abundance in EDF mycoparasites and found that *D.* *cristalligena* subtilase sequences form a group distinct from others, whereas the Zoopagomycotina (*P.* *cylindrospora*, *T.* *sphaerospora* and *S.* *pseudoplumigaleata*) sequences split roughly equally between known proteinase K-like proteins and the group formed by *D.* *cristalligena* (Fig. 6a). Figure 6b shows the domain architecture of orthologous subtilase proteins predicted among the Zoopagomycota and illustrates expansions specific to *D.* *cristalligena*, *T.* *sphaerospora* and *S.* *pseudoplumigaleata*.

### Metallopeptidases in Zoopagomycota

Class M36 metallopeptidases, also known as fungalysins, hydrolyze laminins, elastin, collagen and keratin^46^. The amphibian pathogen *Batrachochytrium dendrobatidis* has an expansion of this metallopeptidase^47^, which is presumably used in the degradation of keratin-rich amphibian skin. Orthologous metallopeptidase proteins were identified in the three species of Zoopagomycotina and *D.* *cristalligena*. A phylogenetic tree (Supplementary Fig. 5) highlights their relationship to other fungalysin proteins and illustrates a unique expansion among the Zoopagomycotina.

### Chitinases in EDF

Chitin is a definitive component of the fungal cell wall^48–50^. It is broken down by chitinases of the glycoside hydrolase family 18 (GH18) and GH19 families and a recently described family (AA11)^51^. The two glycoside hydrolase families do not share similarities in protein sequence, structure or mechanism of action^52^. Furthermore, GH18 chitinases are widely distributed, whereas GH19 chitinases are described mainly from plants and act as defences to insect or fungal invaders.

There are a few known fungal representatives of GH19 chitinases, exclusively from the Cryptomycota and the Microsporidia. We also found GH19 chitinases for the first time in the Chytridiomycota and the Zoopagomycota. A small expansion was found in *Rhizoclosmatium globosum*, a saprotrophic chytridiomycete. Putative GH19 chitinases were also identified in *D.* *cristalligena, Linderina pennispora* and *Coemansia reversa* (Kickxellomycotina), and in *P.* *cylindrospora* (Zoopagomycotina). Only one protein from *D.* *cristalligena* had an additional CBM19 domain (Supplementary Fig. 6). *D.* *cristalligena* also has a unique expansion of the non-catalytic CBM18 family. Finally, the mycoparasites exclusively have *AA11* genes, with clear expansions in *D.* *cristalligena* and *T.* *sphaerospora* (Supplementary Table 4).

### Secondary metabolite expansion in *D.* *cristalligena*

Secondary metabolites are non-essential compounds produced by fungi for various purposes, including antagonism of other microorganisms, pathogenesis and iron chelation. Few secondary metabolites have been identified from EDF^53^. Our results support this observation (Supplementary Table 5), with most of the species containing one or fewer non-ribosomal peptide synthetases (NRPSs) or NRPS-like proteins and two or fewer polyketide synthases or polyketide synthase-like proteins. However, *D.* *cristalligena* possesses 27 *NRPS* genes divided between two lineage-specific expansions in two specific clades (Supplementary Fig. 7). Several of these homologous NRPS proteins also show modular synteny, suggesting multiple duplication events within *D.* *cristalligena*. Based on phylogenetic analysis of adenylation domains (Supplementary Fig. 7), these secondary metabolites are most closely related to epipolythiodioxopiperazine toxins that disrupt cell membranes. *Trichoderma* mycoparasites are known to produce several epipolythiodioxopiperazine toxins, including gliotoxin^54^. Future work is necessary to determine whether these epipolythiodioxopiperazine-like expansions are related to mycoparasitism in *D.* *cristalligena*. Most of the *NRPS* genes reside alone on relatively short contigs (Supplementary Table 6), precluding identification of a traditional fungal secondary metabolite gene cluster.

### Hydrophobins in *C.* *protostelioides*

Hydrophobins are small cysteine-rich proteins involved in the development of aerial hyphae in certain filamentous fungi^55^. These proteins are currently only described in the Dikarya, with no evidence of their presence in EDF^56^. *Caulochytrium* is the only zoosporic genus known to produce aerial stalks and sporangia reminiscent of filamentous fungi^57^. We found 14 putative hydrophobins in the single-cell *C.* *protostelioides* proteome, all of which contained the highly conserved 8-cysteine marker region. Phylogeny and hydropathy profiles both suggest that the *C.* *protostelioides* proteins are more closely related to the group 1 proteins found in the Dikarya (Supplementary Fig. 8). Furthermore, we found one putative group 2 hydrophobin in the *Mortierella elongata* proteome. These findings represent the first examples of hydrophobins outside the Dikarya.

## Discussion

In this study, we used single-cell genomics to create near-complete assemblies of uncultured fungi. This approach allowed us to capture an estimated 73–99% of the genome in multiple-cell co-assemblies ranging from 478 to 8,398 scaffolds. Our analysis shows that genome completeness from a single cell ranges from 6% to 88%, and that combining multiple cells can considerably increase assembly completeness from 6% to 80% (worst case) and from 88% to 96.7% (best case). Co-assemblies of genome data from different single cells further increase genome recovery while losing single-cell resolution for other analyses (for example, heterozygosity).

The co-assemblies allowed annotation of 3,328–12,167 proteins in each species, and common orthologous proteins were used to create a phylogenetic tree, placing these uncultured lineages among their cultured counterparts with robust bootstrap support. We placed Zoopagomycotina as a sister branch to Kickxellomycotina, although with minimal (69% bootstrap) support. A characteristic uniting these subphyla is that certain taxa in both groups produce merosporangia, that is, linear sporangia with few spores^58^. Although *Dimargaris* has been difficult to confidently place in ribosomal RNA gene phylogenies owing to a rapid rate of sequence evolution^59^, here, *D.* *cristalligena* is placed with strong (100%) support as a sister to the Kickxellales. An rRNA-based phylogeny placed *B.* *helicus* with the order Spizellomycetales and Rhizophlyctidiales but without statistical support^60^. In our maximum likelihood tree, *B* *helicus* groups with the Spizellomycetales representative *Spizellomyces* *punctatus*. *Caulochytrium* was placed in the Spizellomycetales by Barr^61^ based on ultrastructural data. However, Barr’s concept of the order has been radically reshaped by molecular phylogenetics^60^. Until this study, no molecular phylogenetic analyses have included *Caulochytrium*; thus, there was no clear null hypothesis for this chytrid. Although *C.* *protostelioides* grouped with the Chytridiales representative *R.* *globosum* (97% support), the precise relationship of *C.* *protostelioides* to other chytrids will require additional genome sequencing.

Single-cell sequencing is uniquely capable of addressing questions in fungal genetics regarding the organization of genetic variation. Analysis of single cells allows testing of whether detected SNPs are specific to an individual or due to variation among multiple genotypes in a variable population. The species targeted by single-cell sequencing were found to correspond to two different groups: non-haploid with high levels of heterozygosity and haploid with negligible heterozygosity (Fig. 3f). Importantly, taxa that we suspected of being diploid had >10,000 SNPs shared across libraries, whereas taxa that were haploid typically had <2,000 SNPs shared across libraries. One caveat is that fungi could be genetically diploid but show low heterozygosity, as is the case for *Saccharomyces* spp.^62^. Surprisingly, we found that *R.* *allomycis* is triploid, which is rare in fungi (for example, in *Epichloë*^63^) and usually reflects a sexually sterile condition. This observation of greater than diploidy has precedent in the microsporidia (for example, the tetraploid *Nosema*^29^), and the chytrid *B.* *dendrobatidis* has diploid to tetraploid nuclei^64,65^. Four of the six basal fungi are not haploid, including three of four Zoopagomycota. The preponderance of heterozygous species at the base of the fungal tree implies a diploid (or higher ploidy) ancestor of the fungi. Beyond estimating ploidy, the single-cell methods presented here could be applied broadly to other fungal taxa where culturing has been unsuccessful (for example, certain rust fungi and ectomycorrhizae), to facilitate genetic mapping, test for genetic segregation and establish mating systems using cohorts of meiotically produced spores.

The uncultured fungi in this study have diverse phylogenetic backgrounds and nutritional strategies. Most are parasites, presumably dependent on hosts for certain nutrients, which may explain their unculturability. By mapping the proteome to primary metabolism pathways, we observed major deficiencies in the ability to synthesize the full set of amino acids and polyamines, which probably result in auxotrophies. Overall, there is a mosaic of losses of essential metabolism genes across the uncultured taxa, consistent with patterns seen in the parasitic Cryptomycota^66^. The deficiencies in the biotin and thiamine pathways in mycoparasites are intriguing given that *Syncephalis* species have been successfully grown using beef liver^67,68^, which contains measurable quantities of both biotin and thiamine^69^. However, *Syncephalis* do not sporulate nor complete their life cycle under these conditions^68^. Most chytrid pollen saprotrophs are culturable, yet the unculturability of *B.* *helicus* may be explained, as it shares deficiencies in spermidine and sulfur metabolism. *M.* *bicuspidata* from *Daphnia* is similar to strain NRRL YB-4993 from brine shrimp (although they are not conspecific^70^) and lacks 15 enzymes that are broadly linked to aspects of urea, sulfate and thiamine metabolism. Given that the majority are also missing from various other target species, this may help to explain its unculturability. *R.* *allomycis* is unlikely to be axenically cultured because it is missing a large number of genes for critical pathways, losses that potentially cannot be corrected for as seen in microsporidia^71^.

Mycoparasitic fungi face unique challenges because they must parasitize a fungal host using fungal enzymes without disrupting their own cells. However, the specificity of this antagonism is not fully characterized^72^. Genomic analysis of the facultative mycoparasite *Trichoderma* spp. revealed several key features, including gene expansions and numerous antifungal-producing secondary metabolite gene clusters^73^. Nevertheless, many fungal lineages contain mycoparasites and there is no generalized analysis of the traits that are selected for in this ecological transition. We did not find any orthologous genes that were a signal of mycoparasitism across all taxa. In obligate mycoparasites, such as those studied here, we found a CAZyme-to-protease ratio more similar to that of animal pathogens rather than plant pathogens. An expansion of subtilases was observed among mycoparasitic Zoopagomycota, forming a group that was distinct from known subtilase families and among subtilases from other fungi. The GH19 chitinases are exclusively restricted to EDF lineages, which suggests that this gene family is ancestral but has been lost.

Having genomes from these EDF enabled us to uncover traits that were previously only described in Dikarya. We observed a vast expansion of secondary metabolism *NRPS* genes unique to *D.* *cristalligena* among other EDF. Similarly, we provide evidence for multiple hydrophobins in *C.* *protostelioides*, the only member of the Chytridiomycota known to form aerial hyphal-like spore-producing structures.

Here, we show that single-cell methods can dramatically expand our understanding of fungal biology. Given the large numbers of as-yet-unsequenced EDF genomes, many of which have never been cultured, these methods help to clarify the expectations for future assembly and functional annotation of genomes from those lineages. Furthermore, given the large numbers of uncultured Dikarya, these methods can be effectively applied across the phylogeny. As the scale of single-cell genomics increases^13^, we will be able to generate a full picture of the fungal tree of life that precisely reveals the complete extent of fungal diversity, regardless of the culturability of the individual taxa.

## Methods

### Strains and sample preparation

A dual culture of parasite *R.* *allomycis* CSF55 with its host fungus *Allomyces* sp. was established and used previously to sequence the genome of the parasite^74^. For this study, spore suspensions of *R.* *allomycis* were obtained under optimal conditions by washing the plates with a dilute Tween solution. An estimated 10^6^–10^7^ spores of the parasite with up to 5% of host spores were obtained. The sample was preserved in 10% sterile glycerol solution, shipped on dry ice and stored at −80 °C.

A dual culture of *C.* *protostelioides* ATCC 52028 with its host *Sordaria* was used to isolate parasitic zoospores at 2.5 × 10^6^ per ml. The zoospore suspension was preserved in 10% dimethylsulfoxide with 10% FBS, shipped on dry ice and stored at −80 °C.

*B.* *helicus* was grown to a high density of cells through enrichment methods using spruce pollen in bog water. The sample was obtained from Perch Pond Fen near Old Town, Penobscot County, Maine, USA, in June 2014. This enrichment culture was filtered through a 40-μm mesh (removing pollen and sporangia) and concentrated by centrifugation to about 5 × 10^4^ zoospores per ml. The sample was preserved in 10% glycerol, shipped on ice and stored at −80 °C.

*M.* *bicuspidata* was isolated from an infected population of the water flea *Daphnia dentifera* grown under laboratory conditions. *D.* *dentifera* samples were rinsed repeatedly with deionized water. Then, insect pins were used to puncture the carapace and a micropipette was used to collect haemolymph, which contained a mixture of *M.* *bicuspidata* yeast cells and ascospores. Cells were preserved in 10% glycerol at a concentration of 10^5^ spores per ml and stored at −80 °C.

*D.* *cristalligena* RSA 468 was grown on V8 juice agar (1 small can of original V8 juice (5.5 oz, 163 ml), diluted to 1 l with deionized water, 3 g CaCO_3_ and 20 g agar) and cultured with *Cokeromyces recurvatus*. Spores were shipped in 10% sterile glycerol.

*S.* *pseudoplumigaleata* Benny S71-1 was grown on *Mucor moelleri* on 10% wheat germ agar (Wg10 (ref. ^75^)). Parasite hyphae and spores were shipped in 50% glycerol.

*T.* *sphaerospora* RSA 1356 was grown on V8 juice agar in dual culture with *C.* *recurvatus* and harvested from petri plates. The sample was stored in 50% glycerol at −80 °C.

*P.* *cylindrospora* RSA 2659 was cultivated on potato dextrose agar with the host *Umbelopsis isabellina*. The culture was grown on many petri dishes and the spores of both the fungus and the host were removed from the culture by washing the plates with 0.2% Triton X-100. An estimated 2.5 × 10^7^ spores per ml of parasite with host were obtained and preserved in 10% glycerol at −80 °C.

All mycoparasites described above are considered obligate mycoparasites. For benchmarking purposes, we used non-single-cell approaches to sequence material gathered from genomic DNA extracted from enrichment cultures of *C.* *protostelioides*. We also took advantage of genomic resources from *M.* *bicuspidata* NRRL YB-4993, a parasite of brine shrimp^70^ related to but not considered conspecific with the *Daphnia* parasitic *M.* *bicuspidata* targeted in this study, and the published genome of *R.* *allomycis*^74^. These three genomic DNA isolates were used to compare with their respective single-cell genomes.

### Single-cell genomics pipeline

The pipeline schema is shown in Supplementary Fig. 1. After sample collection, as described in the preceding section, individual cells were isolated using a one-step or two-step FACS (BD Influx Cell sorter) procedure. Target population enrichment in the original sample, determined by microscopy, dictated the use of one-step or two-step FACS. Cells were sorted into 384-well plates at counts of 1, 10, 30, 50 or 100 cells per well. Sorting accuracy was verified microscopically (Zeiss Axio Observer D1) and according to morphology.

Verified target cells were lysed using a combination of enzymatic and alkaline solutions. Briefly, cell lysis solutions were prepared with the following reagents: KOH dry pellets (reconstituted to 500 mM with nuclease-free water), 1 M dithiothreitol, and HCl (Stop Buffer) were obtained from the REPLI-g WGA Single-Cell Kit (150345, Qiagen); Tween 20 (P9416-100 ml, Sigma)); 0.5 M EDTA (AM92606, Ambion); proteinase K (P8107S, NEB); phenylmethylsulfonyl fluoride (532789-5 g, Sigma); and EGTA (E3889-10 g, Sigma). Genome amplification via multiple displacement amplification was achieved using either the REPLI-g WGA Single-Cell Kit (150345, Qiagen) according to the manufacturer’s instructions, or in-house using 10 mM dNTP (N0447L, NEB), 500 mM hexamers (37617009, IDT), phi29 polymerase (M029L, NEB), DMSO (D8418-50 ml, Sigma) and 10× buffer (400 mM Tris-HCl, pH 7.5 (AM9855, Ambion), 500 mM KCL (AM9640G, Ambion), 100 mM MgCl_2_ (AM9530, Ambion), 50 mM (NH_4_)_2_SO_4_ (AA4418-100 g, Sigma) and 20 mM dithiothreitol (P2325, Invitrogen)). We determined that, although the in-house approach is cheaper and yields better coverage, it is less practical for large-scale production as it requires several days of reagent cleaning prior to the reaction, whereas REPLI-g comes pre-purified and requires only a few hours of additional cleaning. Furthermore, downstream co-assembly of libraries reduces the problem of random bias.

As a quality-control step, amplified cells were 18S rDNA screened using the following primers: M13CRYPTO2-2F (5′-GTTTTCCCAGTCACGACCACAGGGAGGTAGTGACAG-3′), M13AU4v2 (5′-CAGGAAAAGCTATGACGCCTCACTAAGCCATTC-3′), M13DPD360FE (5′-GTTTTCCCAGTCACGACCGGAGARGGMGCMTGAGA-3′), M13DPD1492RE (5′-CAGGAAACAGCTATGACACCTTGTTACGRCTT-3′), M13SR1RFor (5′-GTTTTCCCAGTCACGACTACCTGGTTGATYCTGCCAGT-3′) and M13NS4Rev (5′-CAGGAAACAGCTATGACCTTCCGTCAATTCCTTTAAG-3′). Amplified 18S rDNA fragments were treated with ExoSap-IT (Thermo Fisher Scientific) and Sanger sequenced. Sequences were queried using BLAST against the National Center for Biotechnology Information non-redundant (NCBI nr) and Assembling the Fungal Tree Of Life (AFTOL)^76^ databases. Verified target wells were prepared using the TruSeq library method. Between three and seven libraries were pooled together and sequenced on one lane of Illumina HiSeq 1T v4-v6 2 × 150 bp. All pooled libraries belonged to the same species. Prior to establishing this protocol, we tested for crosstalk between TruSeq libraries on the MiSeq platform during pre-assembly library screening and found no such occurrence for the fungal single-cell pipeline. However, owing to previous observations of library crosstalk in the Joint Genome Institute (JGI) bacterial single-cell pipeline, the fungal single-cell pipeline decided on the cautionary same-species pooling until further examination was completed. Fungal single-cell pipeline TruSeq libraries were prepared on eight tube strips with individual caps using automated multichannel pipettes, which excludes plate-based robotic potential cross-contamination between samples.

In the fungal single-cell pipeline, there are several steps of rigorous filtering of potential contaminating sequences, at both the read level (pre-assembly) and the contig level (post-assembly). All Illumina reads were run through a filtering pipeline prior to assembly. BBDuk (https://sourceforge.net/projects/bbmap/) was used with the settings filterk = 27 and trimk = 27 to remove Illumina adapters, known Illumina artefacts, phiX and quality trim both ends to Q12. Resulting reads containing more than one ‘N’, with quality scores (before trimming) averaging <8 over the read or length under 40 bp after trimming were discarded. Remaining reads were mapped to a masked version of human hg19 with BBMap, discarding all hits over 93% identity. Reads mapping to mouse were also similarly discarded. Reads were also normalized to make the coverage more uniform, including removing reads that have very high coverage (>100). Individual libraries from each species were assembled with SPAdes^77^ (v2.6 or v3.0), using the ‘careful’ and ‘single-cell’ options, and k-mer sizes of 21, 33 or 55. After assembly, scaffolds of <2 kb in length were removed as, in our experience, they are phylogenetically ambiguous. Assembled contigs were also compared using BLAST to a set of contaminant databases: bacterial, mouse, human, feline and canine. Finally, we performed tetramer principle component analysis and removed any outlier contigs. This extra cautious read usage for assembly probably leads to reduced completeness and removes symbiotic occurrences but guarantees one species genome instead.

Supplementary Table 1 presents summaries of HiSeq libraries of variable number of cells for each organism. Bold libraries denote inclusion in co-assemblies. Underlined libraries denote individual annotation. To generate co-assemblies, reads from each library were extracted, pooled and co-assembled, again with SPAdes. The final co-assemblies were annotated using the JGI Annotation Pipeline^78^. For *D.* *cristalligena*, *C.* *protostelioides*, *R.* *allomycis* and *M.* *bicuspidata*, all individual libraries were additionally annotated. For *P.* *cylindrospora*, *T.* *sphaerospora*, *S.* *pseudoplumigaleata* and *B.* *helicus*, only the most complete (highest CEGMA score) 1-cell and 100-cell (1 cell and 50 cell for *T.* *sphaerospora*) individual libraries were additionally annotated. All final annotations were loaded into MycoCosm, the JGI Fungal Genomics Resource^78^, for public presentation and comparative analysis.

### Annotation completeness

CEGMA^26^ as a general measure of completeness has been deprecated in favour of BUSCO^79^. However, because the underlying data for BUSCO relies heavily on Dikarya fungi, we have observed (Supplementary Fig. 9) that BUSCO dramatically underestimates the coverage of EDF lineages, particularly within the Chytridiomycota, Blastocladiomycota and Mucoromycota. As such, we continue to use CEGMA metrics for this study as it focuses primarily on EDF.

### Heterozygous polymorphism discovery

Paired-end reads were aligned to assembled draft genomes using BWA^80^ (v0.7.12-r1044) using default parameters. Variants were identified using FreeBayes^81^ (v1.0.2-58-g054b257) with parameters ‘–pooled-continuous –min-coverage 5’. As non-haploid organisms have multiple genome copies per cell, increased counts of higher-frequency SNPs (allele frequency > 0.25) in their single cells are indicative of heterozygosity. Only SNPs with an allele frequency >25% were kept for further analysis. Comparisons of ploidy levels between species were performed on four single-cell libraries with the highest completeness and quality. K-mer graphs were plotted using the kmercountexact.sh script of the BBTools package (http://jgi.doe.gov/data-and-tools/bbtools/).

For intraspecific variation, reads from the eight *D.* *cristalligena* one-cell libraries were aligned to the *D.* *cristalligena* co-assembled genome using BBMap (v37.76) (https://sourceforge.net/projects/bbmap/). Variants were identified using FreeBayes^81^ (v1.1.0-54-g49413aa) with parameters ‘–-min-coverage 5 –F 0.01 –C 2 –no-mnps –no-complex’. SNPs with 100% disagreement with reference and 100% frequency were kept and mapped to genes for which all libraries had >90% coverage. A set visualization plot (Supplementary Fig. 2) was generated using the R implementation of the UpSet set visualization technique^82^ (https://cran.r-project.org/package=UpSetR).

### Functional analyses

Subtilase cluster analysis was achieved using CLANS^83^ and a list of identified protein sequences described in Li et al.^45^. Metallopeptidase and hydrophobin trees were constructed using RAxML^84^ (v8.2.2; GAMMA + WAGF substitution model; 100 bootstrap replicates) and PFAM seed set sequences: PF02128 and PF01185 + PF06766, respectively. Trees were visualized using the Interactive Tree of Life (iTOL) website^85^ (http://itol.embl.de).

For phylogenetic analysis of identified *NRPS* genes, adenylation domains were mined from proteomes using HMMER^86^ (v3.1b2) with a hidden Markov model based on the fungal and bacterial domains identified by Bushley and Turgeon^53^. MUSCLE^87^ (v3.8.31) was used to align extracted domains with those used for the hidden Markov model creation and gaps were removed manually. RAxML^84^ (v8.2.8) was used for phylogenetic reconstruction using the gamma model of rate heterogeneity and the RTREV substitution matrix with 100 bootstrap replicates.

### Phylogenetic analysis

All-v-all blastp^88^ (v2.2.26) was run using a cut-off of 1 × 10^−5^ on the predicted proteomes of the representative set of fungal and outgroup species provided in Supplementary Table 2. Clusters were predicted using MCL^89^ (v1.008) with an inflation value of 2. A python script identified clusters containing at most one gene copy per genome, allowing for up to 8 missing taxa per cluster, which resulted in 805 total clusters selected. Each cluster was aligned using MAFFT^90^ (v7.047) on each cluster, with a multiple sequence alignment algorithm detected automatically (using the –auto flag). Alignments were trimmed with Gblocks^91^ (v0.91b) and concatenated, followed by rapid bootstrap maximum likelihood tree building with RAxML^84^, 27,751 distinct alignment patterns, 100 bootstrap replicates and using the GAMMA + WAGF protein model. For all 29,255 positions in the alignment, the target genomes had a median of 14% missing, compared to the median of 2.5% missing of others.

### Orthogroup reconstruction

Using MCL clustering^89^, orthologues were collected using a minimum of three taxa per orthogroup, ignoring single-cell *C.* *protostelioides* and *R*. *allomycis* in favour of the respective co-assemblies. The R package APE^92^ was used for ancestral-state reconstruction. Protein functions predicted using PRIAM^93^ were mapped to each internal node of the phylogenetic tree presented in Fig. 1. Gains and losses in single-cell lineages were considered as relative to the ancestral fungal node (Supplementary Fig. 3): *D.* *cristalligena* shows noticeable gains in all high-level metabolic categories, whereas *B.* *helicus* has mainly losses.

### Metabolic reconstruction

Enzyme classifications were predicted for all 60 proteomes in Supplementary Table 2 using PRIAM^93^. A subset of 24 fungal, non-single-cell proteomes were defined as ‘free living’ based on culturability. The presence/absence counts were converted to a binary presence/absence matrix for each enzyme classification found in each species and filtered according to ‘found in free-living’ threshold of present in 18 out of 24 (75%) free-living species and absent in at least 5 single-cell species. ‘Single cell’ includes *C.* *protostelioides* and *R.* *allomycis* culture isolate genomes in place of those respective single-cell genomes. The resulting pattern of conserved losses among ‘core’ metabolic pathways was summarized and displayed using IPath^94^.

### Reporting Summary

Further information on research design is available in the Nature Research Reporting Summary linked to this article.

## Supplementary information

Supplementary Information Supplementary Methods, Supplementary Notes, Supplementary References, Supplementary Tables 5–8, Supplementary Figures 1–9 and Supplementary Dataset legends.

Reporting Summary

Supplementary Table 1 Select statistics for all sequenced libraries from each target species.

Supplementary Table 2 Descriptions and sources for fungal and outgroup proteomes used in comparative analyses.

Supplementary Table 3 Summary of tree phylogenies generated using select individual libraries.

Supplementary Table 4 Abundance counts of different chitinase and related CAZyme families among the target genomes.

Dataset 1 Contains 6 phylogenetic trees, each using a different library of *Caulochytrium protostelioides*: one 1-cell, one 10-cell, and four 100-cell libraries.

Dataset 2 Contains 14 phylogenetic trees, each using a different library of *Dimargaris cristalligena*: eight 1-cell, one 50-cell, and five 100-cell libraries.

Dataset 3 Contains 5 phylogenetic trees, each using a different library of *Metschnikowia bicuspidata*: three 1-cell, one 10-cell, and one 100-cell libraries.

Dataset 4 Contains 14 phylogenetic trees, each using a different library of *Rozella allomycis*: eleven 1-cell and three 100-cell libraries.

Dataset 5 Contains 1 phylogenetic tree, built using the co-assemblies and presented graphically as Figure 1.

## Data Availability

The co-assembled genomes and annotations of the target species are available through MycoCosm (https://genome.jgi.doe.gov/fungi) and Genbank using the following MycoCosm URLs and NCBI accessions, respectively: *R.* *allomycis* CSF55 single cell (https://genome.jgi.doe.gov/Rozal_SC1; QUVT00000000), *B.* *helicus* Perch Fen single cell (https://genome.jgi.doe.gov/Blyhe1; QPFV00000000), *C.* *protostelioides* ATCC 52028 single cell (https://genome.jgi.doe.gov/Caupr_SCcomb; QUVS00000000), *D.* *cristalligena* RSA 468 single cell (https://genome.jgi.doe.gov/DimcrSC1; QRFA00000000), *P.* *cylindrospora* RSA 2659 single cell (https://genome.jgi.doe.gov/Pipcy3_1; QPFT00000000), *T.* *sphaerospora* RSA 1356 single cell (https://genome.jgi.doe.gov/Thasp1; QUVU00000000), *S.* *pseudoplumigaleata* Benny S71-1 single cell (https://genome.jgi.doe.gov/Synps1; QUVV00000000) and *M.* *bicuspidata* single cell (https://genome.jgi.doe.gov/Metbi_SCcomb; QUVR00000000). The whole-genome sequence for the non-single-cell isolate *C.* *protostelioides* ATCC 52028 is available through MycoCosm (https://genome.jgi.doe.gov/Caupr1) and Genbank (QAJV00000000). The whole-genome sequences for the non-single-cell isolate of *R.* *allomycis* CSF55 was not determined in this study and is available through MycoCosm (https://genome.jgi.doe.gov/Rozal1_1) and Genbank (ATJD00000000). The genome sequence for the non-single-cell *M.* *bicuspidata* NRRL YB-4993 was also not determined in this study and is available through MycoCosm (https://genome.jgi.doe.gov/Metbi1) and Genbank (LXTC00000000).
