# Supplementary material for: Leveraging single-cell genomics to expand the fungal tree of life
Source: Nat Microbiol. 2018 Oct 8;3(12):1417–28. doi: 10.1038/s41564-018-0261-0 (PMC6784888; doi:10.1038/s41564-018-0261-0)
Supplement: Supplementary file 1 — Supplementary Methods, Supplementary Notes, Supplementary References, Supplementary Tables 5–8, Supplementary Figures 1–9 and Supplementary Dataset legends. [file 41564_2018_261_MOESM1_ESM.pdf]

nature microbiology

SUPPLEMENTARY INFORMATION

https://doi.org/10.1038/s41564-018-0261-0

In the format provided by the authors and unedited.

Leveraging single-cell genomics to expand the fungal tree of life

Steven R. Ahrendt[1,2], C. Alisha Quandt[3,9], Doina Ciobanu[1], Alicia Clum[1], Asaf Salamov[1], Bill Andreopoulos[1], Jan-Fang Cheng[1], Tanja Woyke[1], Adrian Pelin[4], Bernard Henrissat[5,6,7], Nicole K. Reynolds[8], Gerald L. Benny[8], Matthew E. Smith[8], Timothy Y. James[3]* and Igor V. Grigoriev[1,2]*

[1]US Department of Energy Joint Genome Institute, Walnut Creek, CA, USA. [2]Department of Plant and Microbial Biology, University of California Berkeley, Berkeley, CA, USA. [3]Department of Ecology and Evolutionary Biology, University of Michigan, Ann Arbor, MI, USA. [4]Ottawa Hospital Research Institute, Centre for Innovative Cancer Research, Ottawa, Ontario, Canada. [5]Architecture et Fonction des Macromolécules Biologiques, UMR 7857 CNRS, Aix-Marseille University, Marseille, France. [6]Institut National de la Recherche Agronomique, USC 1408 Architecture et Fonction des Macromolécules Biologiques, Marseille, France. [7]Department of Biological Sciences, King Abdulaziz University, Jeddah, Saudi Arabia. [8]Department of Plant Pathology, University of Florida, Gainesville, FL, USA. [9]Present address: Department of Ecology and Evolutionary Biology, University of Colorado Boulder, Boulder, CO, USA. *e-mail: tyjames@umich.edu; ivgrigoriev@lbl.gov

SUPPLEMENTARY INFORMATION

Leveraging single-cell genomics to expand the Fungal Tree of Life

Steven R. Ahrendt[1,2], C. Alisha Quandt[3†], Doina Ciobanu[1], Alicia Clum[1], Asaf Salamov[1], Bill Andreopoulos[1], Jan-Fang Cheng[1], Tanja Woyke[1], Adrian Pelin[4], Bernard Henrissat[5,6,7], Nicole K. Reynolds[8], Gerald L. Benny[8], Matthew E. Smith[8], Timothy Y. James[3*], and Igor V. Grigoriev[1,2*]

[1] US Department of Energy Joint Genome Institute
Walnut Creek, CA, 94598, USA

[2] Department of Plant and Microbial Biology,
University of California Berkeley, Berkeley, CA, 94720, USA

[3] Department of Ecology and Evolutionary Biology,
University of Michigan, Ann Arbor, MI, 48109, USA

[4] Ottawa Hospital Research Institute,
Centre for Innovative Cancer Research, Ottawa, K1H 8L6, Canada

[5] Architecture et Fonction des Macromolécules Biologiques, UMR 7857 CNRS
Aix-Marseille University, F-13288 Marseille, France

[6] Institut National de la Recherche Agronomique
USC 1408 Architecture et Fonction des Macromolécules Biologiques, F-13288 Marseille, France

[7] Department of Biological Sciences
King Abdulaziz University, Jeddah, Saudi Arabia

[8] Department of Plant Pathology
University of Florida, Gainesville, FL, 32611, USA

[†] Current address:
Department of Ecology and Evolutionary Biology
University of Colorado Boulder, Boulder, CO, 80309, USA

*Corresponding Authors:
Timothy Y. James, tyjames@umich.edu
Igor V. Grigoriev, ivgrigoriev@lbl.gov

Supplementary Methods

Axenic culture experiments on the mycoparasitic fungi *Dimargaris cristalligena, Piptocephalis cylindrospora* and *Syncephalis pseudoplumigaleata*

Culture materials – The genome-sequenced isolates of *Dimargaris cristalligena* RSA 468 (host: *Cokeromyces recurvatus*), *Piptocephalis cylindrospora* RSA 2659 (host: *Umbelopsis isabellina*), and *Syncephalis pseudoplumigaleata* S71 (host: *Cunninghamella* sp.) were grown in co-culture with their hosts on V8 or CV8 media supplemented with antibiotics. Culture material for *Thamnocephalis sphaerospora* RSA 1356 was not available. *D. cristalligena* was grown from lyophilized culture sealed in glass. This vial was scored with a file, broken, and the dried tissue was placed in brain-heart infusion broth. When hyphae were seen growing in the broth, the tissue was transferred to V8 media using a sterile pipette. Cultures were kept in an 18˚C incubator.

Media recipes – Five supplements were tested for their effect on axenic culturing of mycoparasites based on the results of genome sequencing: a trio of amino acids (L-cysteine, L-methionine, L-tryptophan), biotin, magnesium sulfate, thiamine, and spermidine. Based on previous studies on axenic culturing of *P. virginiana* (= *P. cylindrospora*)[94], *D. bacillispora*, *D. verticellata*[95], and *D. xerosporica*[96], malt extract-yeast extract (MEYE) medium was used as the base for all recipe variations. All of these studies produced only limited growth of the target fungi but the most successful attempts all used MEYE or a similar medium. For our experiments, seven different media recipes were tested: 1) a negative control consisting of MEYE without any additional supplements, 2) a complete treatment with all five supplements added, and 3) five recipes each missing a different supplement compared to the complete treatment (Supplementary Table 7). No antibiotics were used in any of these recipes. Biotin, thiamine, and spermidine were all added after autoclaving the media to avoid heat degradation. All media were prepared in 250mL volumes, autoclaved for 20 minutes, and poured into sterile 60 x 15mm petri dishes. The MEYE base was 0.75 g malt extract, 0.75 g yeast extract, 1.25 g peptone, 2.5 g dextrose, 4.5 g agar, and distilled water to fill a total volume of 250 mL. The supplements were added in the following amounts: 0.15 g $MgSO_4$ $7H_2O$, 0.05 g of the three amino acids, 0.025 g thiamine, 0.025 g biotin, and 12 µL of 98% pure spermidine.

Culture isolation – Once sufficient growth of the parasites on their hosts was observed, sterile transfer of parasite spore material to the experimental media (without a host present) was performed using one of two techniques. In the first technique, a fine nichrome wire was flame sterilized and touched against sporangia in order to coat the wire with spores or to break sporophores away from the host material. This technique was used for *P. cylindrospora* and *S. pseudoplumigaleata* due to their close proximity to host sporangia and potential for host contamination. The spores or sporophores were then inoculated onto the clean plates with a gentle stab into the agar. For the second method, a flame sterilized spatula tool was used to cut a small piece of agar from the experimental plate and then touched to the sporangia to adhere the spores to the agar chunk. The chunk with the spores was then placed back onto the clean plate. This technique was used for *D. cristalligena* because it is wet-spored and the sporophores are much taller than the host. Two transfers were made for each species per treatment for a total of 56 plates. Cultures were stored in an 18˚C incubator. Plates were inspected every other day for fungal growth and contamination.

Supplementary Notes

Piptocephalis cylindrospora – The parasite grew well and produced numerous sporangia when grown on the host fungus. However, as was found previously[94], *P. cylindrospora* had limited axenic growth on MEYE media on all treatments. The sporophores were dwarfed and depauperate and the colonies remained small without growing beyond the initial inoculation point. Spores were produced in highly reduced numbers compared to those grown on a host. Slightly reduced growth was observed on media lacking thiamine and spermidine, but otherwise the treatments all produced similarly sized colonies (including the MEYE control). The plates of the fully supplemented media (e.g. all supplements added) were contaminated with bacteria which likely inhibited the growth of *P. cylindrospora*. We found that *P. cylindrospora* did not survive transfer in axenic culture on the fully supplemented media. Manocha[94] also reported that *P. cylindrospora* died in axenic culture after one or two transfers.

Dimargaris cristalligena – Isolate RSA 468 grew well from the lyophilized material at 18˚C and produced copious sporangia on the host. In axenic culture, hyphae were dwarfed and only limited growth was observed. Within the first week of growth, the fully supplemented media supported the most growth of *D. cristalligena.* Very limited growth was seen on the treatment lacking biotin but no growth was observed on the other treatments. A few plates from various treatments were contaminated with bacteria. After three weeks, limited growth was observed on all treatments except for the control and the treatment lacking $MgSO_4$ (Supplementary Figure 4). It is not clear whether the lack of growth was due to the media formulation or inability of the spores to germinate.

Syncephalis pseudoplumigaleata – No *S. pseudoplumigaleata* grew on any media variations. In some cases plates were contaminated with host fungi or bacteria so the treatments could not be compared. In co-culture with the host, *S. pseudoplumigaleata* grew and sporulated abundantly.

Supplementary Discussion

None of the fungi tested in these assays were able to complete their lifecycle in any of the media formulations, suggesting that additional stimuli or co-factors may be present on or inside the hyphae of host fungi that are required by the parasites. The case of *D. cristalligena* showed the most promise whereby the fully supplemented media clearly allowed for the most rapid and abundant growth when compared with the other treatments. This suggests that further explorations into the genome and metabolism of these fungi may yield additional clues and allow for additional supplement experiments that could be successful in the future.

A number of previous studies have attempted to grow mycoparasitic EDF in culture and met with limited results. Many of these fungi can be transferred and grown in small, slow growing colonies on highly enriched media but they rarely or never sporulate and they typically cannot be transferred to a new media plate. In the case of some *Syncephalis* species, they can be grown more effectively on high protein substrates such as beef liver media but they do not sporulate. Therefore, none of these fungi can complete their lifecycles without their host fungi. Below we provide a brief synopsis of experiments that have been performed previously and information that was obtained from these culture experiments.

Several isolates of *Syncephalis* (including *S. nodosa, S. sphaerica* and *S. plumigaleata*) have been grown axenically on beef liver media[97,98]. *Dispira cornuta* also grew axenically on scrambled eggs, beef, and swordfish[99]. Three species of *Dimargaris* (*D. verticellata, D.*

bacillispora, and *D. xerosporica*) grew axenically on supplemented media[95,96]. For all three species, glycerol was a more effective carbon source than other sugars. *Dimargaris verticellata* and *D. bacillispora* grew better on media containing added thiamine than when it was not included in the media[95]. The addition of biotin, amino acids, and other vitamins had no effect on the growth of *D. verticellata* or *D. bacillispora*, but a moderate increase in growth was observed for *D. xerosporica* when L-asparagine, L-alanine, and L-glutamine were added at 5g/L[96]. The axenic cultures also were very slow growing, taking several weeks to obtain sizable colonies.

Piptocephalis virginiana (= *cylindrospora*) has been grown axenically on MEYE media[94,100]. The sporophores were dwarfed and depauperate and the fungus did not survive more than one or two transfers in axenic conditions (e.g. similar to the observations made in this study). Manocha[94] found that spores harvested from axenic cultures of *P. virginiana* lacked gamma linolenic acid and differed in their overall lipid content compared to spores produced when grown on a host. Manocha[94] suggested that the lack of lipids in the axenic spores contributed to their failure to germinate and grow in the absence of a host.

Supplementary References

94. Manocha, M. S. Host-Parasite Relations in a Mycoparasite (III). Morphological and Biochemical Differences in the Parasitic- and Axenic-Culture Spores of *Piptocephalis virginiana*. *Mycologia* **67,** 382–391 (1975).
95. Barnett, H. L. Nutritional Requirements for Axenic Growth of Some Haustorial Mycoparasites. *Mycologia* **62,** 750–760 (1970).
96. Joshi, M. C., Rani, S. & Mehrotra, B. S. Nutritional requirements for axenic growth of a haustorial mycoparasite, *Dimargaris xerosporica* (Mehrotra and Baijal) Benjamin. *Natl. Acad. Sci. Lett. - India* **6,** 285–287 (1983).
97. Ellis, J. J. On growing *Syncephalis* in pure culture. *Mycologia* **58,** 465–469 (1966).
98. Lazarus, K. L., Benny, G. L., Ho, H.-M. & Smith, M. E. Phylogenetic systematics of *Syncephalis* (Zoopagales, Zoopagomycotina), a genus of ubiquitous mycoparasites. *Mycologia* **109,** 333–349 (2017).
99. Ayers, T. T. Growth of Dispira cornuta in Artificial Culture. *Mycologia* **25,** 333–341 (1933).
100. Berry, C. R. & Barnett, H. L. Mode of Parasitism and Host Range of *Piptocephalis virginiana*. *Mycologia* **49,** 374–386 (1957).

Supplementary Table 5. Counts of different classes of secondary metabolism genes among target genomes

Isolate	NRPS	NRPS-like	PKS	PKS-like	Hybrid NRPS-PKS	DMAT	Total
D. cristalligena	21	7	0	2	0	0	30
S. pseudoplumigaleata	1	0	0	1	0	0	2
B. helicus	0	1	0	2	0	0	3
T. sphaerospora	0	1	0	1	0	0	2
M. bicuspidata	0	1	0	2	0	0	3
P. cylindrospora	0	0	0	0	0	0	0
C. protostelioides	0	0	0	0	0	0	0

Supplementary Table 6. Details of NRPS and NRPS-like genes identified in *Dimargaris cristalligena*

Protein Model	Contig	# of A domains	Length of Contig	Flanking homology?
DimcrSC1_36903	Node_41	6	59150	Yes
DimcrSC1_38103	Node_595	3	17469	No
DimcrSC1_7858	Node_193	1	33906	Yes
DimcrSC1_40295	Node_209	3	32785	Yes
DimcrSC1_36326	Node_143	1	39536	No
DimcrSC1_37012	Node_8	3	88073	Yes
DimcrSC1_34052	Node_541	3	18959	No
DimcrSC1_35489	Node_575	2	17968	No
DimcrSC1_40741	Node_720	1	14908	No
DimcrSC1_39407	Node_1130	2	8409	No
DimcrSC1_40580	Node_702	3	15202	No
DimcrSC1_39455	Node_1155	2	8099	No
DimcrSC1_22007	Node_1226	1	7312	No
DimcrSC1_37587	Node_785	1	13448	No
DimcrSC1_28565	Node_974	1	10472	Yes
DimcrSC1_40022	Node_872	1	11902	No
DimcrSC1_40310	Node_333	1	26502	Yes
DimcrSC1_35934	Node_1528	1	4813	Yes
DimcrSC1_35539	Node_567	2	18259	No
DimcrSC1_35376	Node_655	1	16116	No
DimcrSC1_22401	Node_1288	1	6701	No
DimcrSC1_35250	Node_1660	1	3941	No
DimcrSC1_41021	Node_1379	1	5774	No
DimcrSC1_3480	Node_504	1	20050	Yes
DimcrSC1_6602	Node_734	1	14751	No
DimcrSC1_35697	Node_932	1	10954	No

Supplementary Table 7. Supplements used in each of seven different experimental media recipes.

**Treatment**	**Supplements included**
Control	None
Complete	Biotin, L-cysteine, L-methionine, L-tryptophan, magnesium sulfate, thiamine, spermidine
1	Biotin, magnesium sulfate, thiamine, spermidine
2	L-cysteine, L-methionine, L-tryptophan, magnesium sulfate, thiamine, spermidine
3	Biotin, L-cysteine, L-methionine, L-tryptophan, thiamine, spermidine
4	Biotin, L-cysteine, L-methionine, L-tryptophan, magnesium sulfate, spermidine
5	Biotin, L-cysteine, L-methionine, L-tryptophan, magnesium sulfate, thiamine

Supplementary Table 8. Results of axenic growth of mycoparasite fungi on different media formulations.

Isolate	Control	Complete	No aminos	No biotin	No thiamine	No spermidine	No $MgSO_4$
P. cylindrospora	++	contam	++	++	+	+	++
D. cristalligena	0	++	+	+	+	+	0
S. pseudoplumigaleata	0	0	contam	contam	contam	contam	contam

0= no growth
"contam" = contamination of agar plates, either with host fungi or bacteria
+ = growth was observed
++ = faster and/or more abundant growth (relative to other treatments)

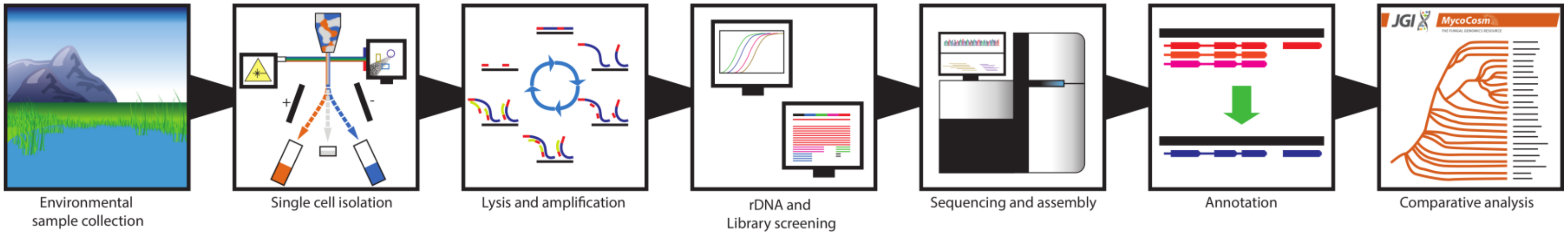

Supplementary Figure 1. Single-cell workflow schematic. Workflow diagram summarizing the seven steps involved in eukaryotic single-cell sequencing: environmental sample collection from aquatic or soil environments; isolation using fluorescence activated cell sorting (FACS); multiple displacement amplification (MDA); library screening using rtPCR and BLAST; Illumina sequencing; annotation using the JGI Annotation Pipepline; and comparative analysis using Mycocosm.

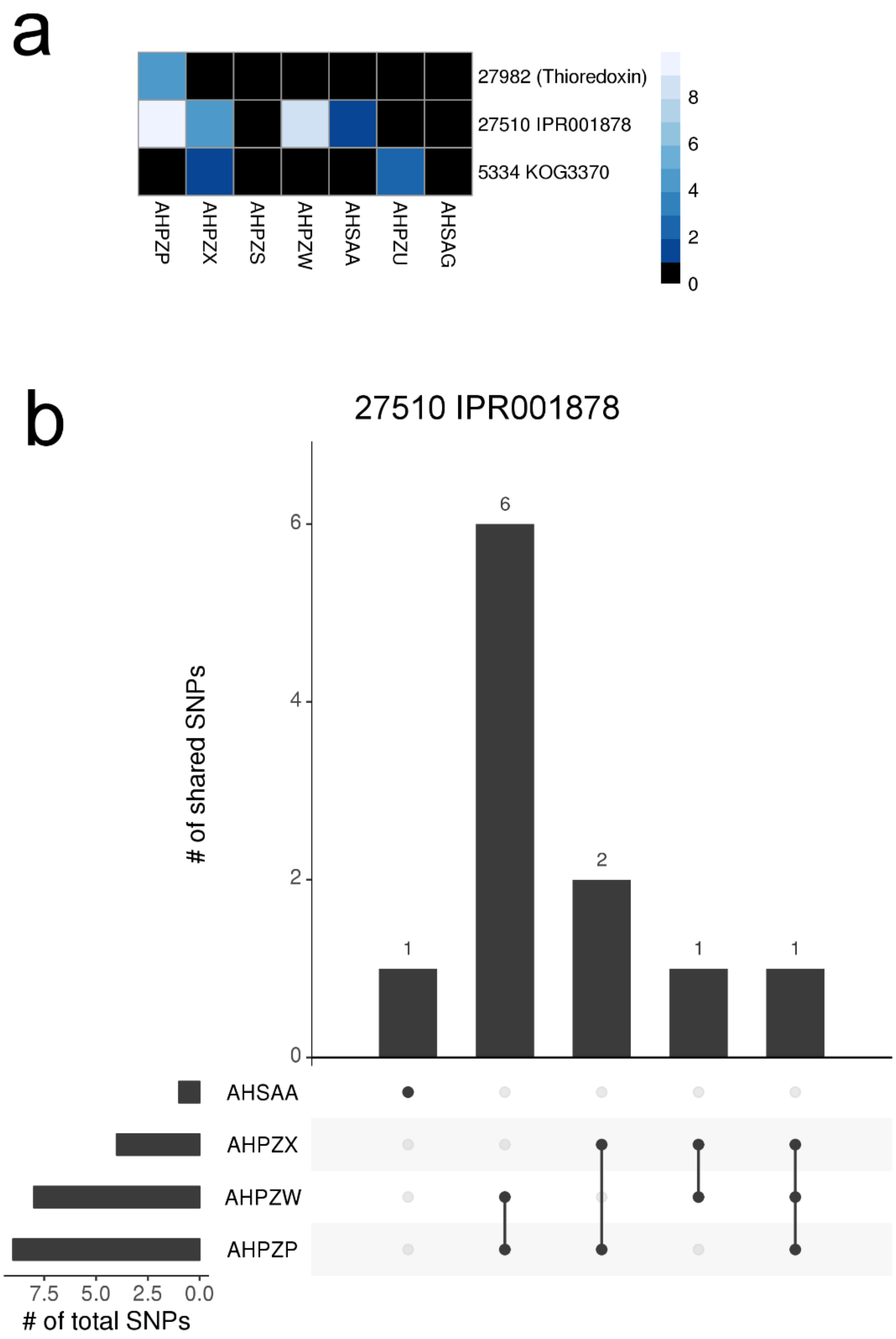

Supplementary Figure 2. lntraspecific SNP variation in *D. cristalligena*. a) Heatmap of counts of individual SNPs per gene in a given 1-cell library. Genes are annotated with corresponding Mycocosm protein ID and functional annotation (if available), with IDs from either KOG, PFAM, or lnterpro, or best blast hit if IDs are unavailable. b) Upset intersection diagram categorizing shared and unique SNPs between libraries for genes corresponding to protein 27510.

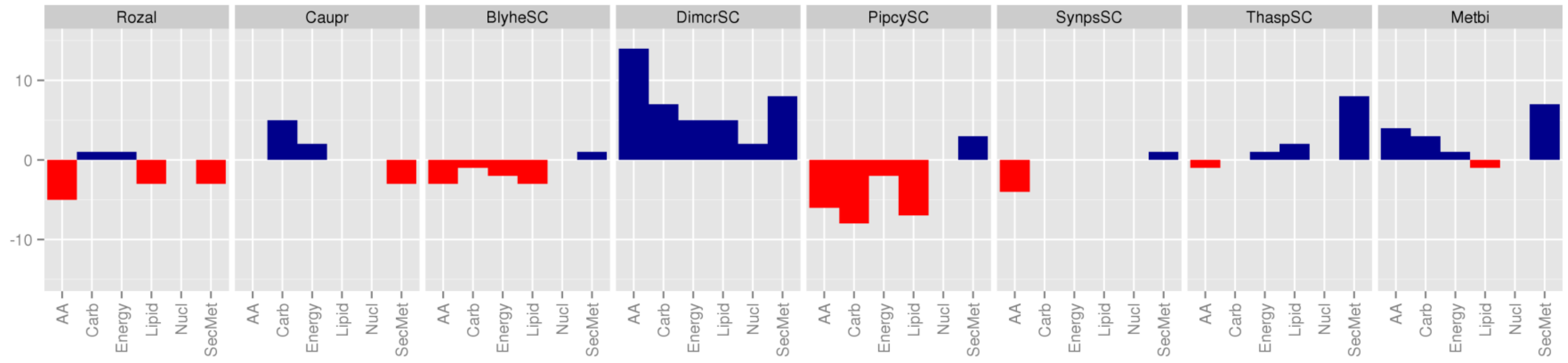

Supplementary Figure 3. Orthogroup differences between fungal ancestor and single-cell lineages. Each orthogroup derived from MCL clustering data was assigned EC designation using PRIAM. Enzymes belonging to high-level metabolic pathways were clustered for each single-cell lineage, and compared to the pathways present in the fungal ancestor. Negative values (red) represent losses and positive values (blue) represent gains of high-level metabolic pathways. AA=Amino Acid; Carb=Carbohydrate; Nucl=Nucleic Acid; SecMet=Secondary Metabolism

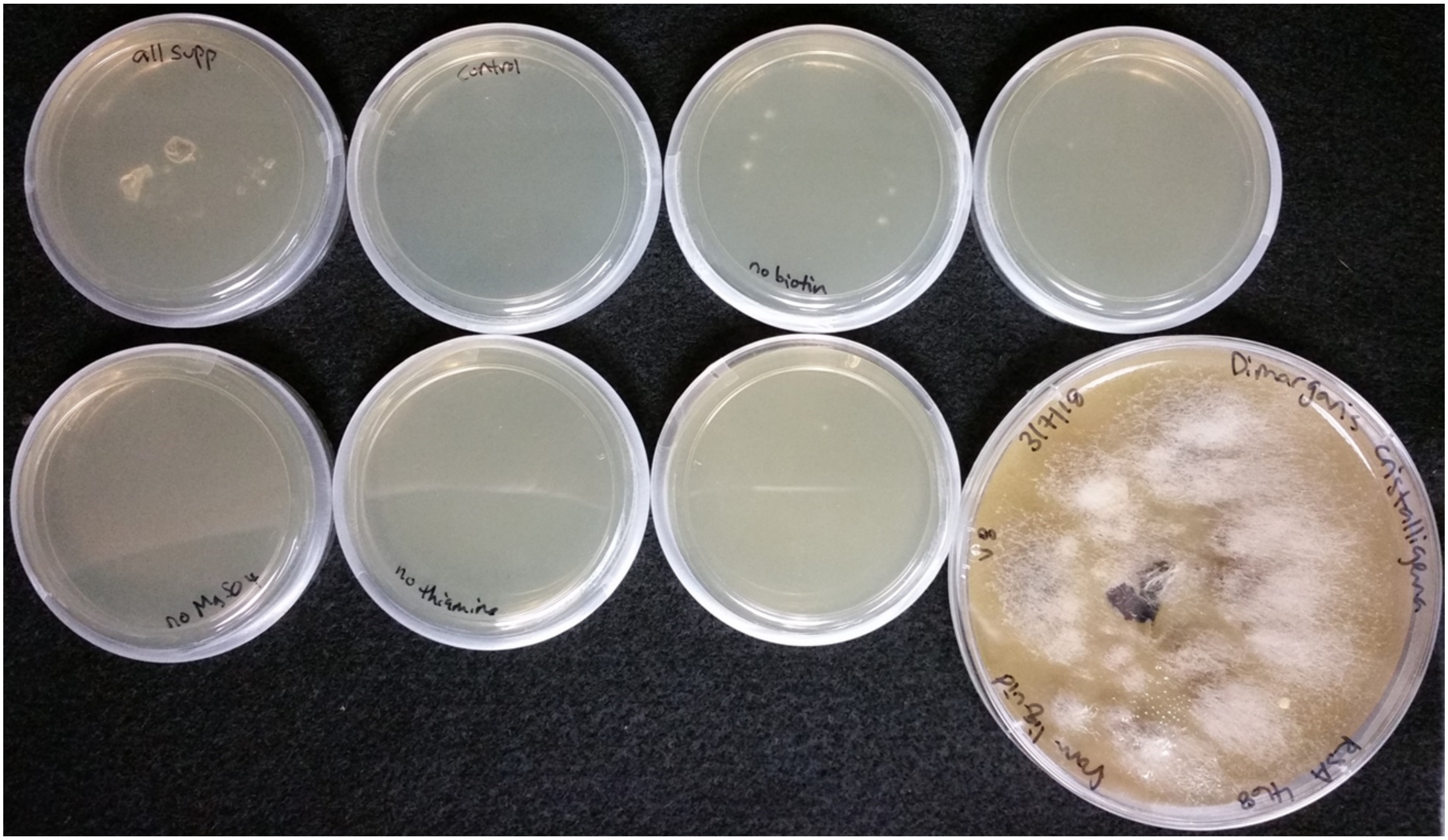

Supplementary Figure 4. Axenic growth of *Dimargaris cristalligena* RSA 468 on seven different media formulations. The complete media recipe comprised malt extract-yeast extract (MEYE) with biotin, L-cysteine, L-methionine, L-tryptophan, magnesium sulfate, thiamine, and spermidine added. The control recipe contained MEYE with no added supplements. The remaining treatments consisted of the complete recipe with one supplement subtracted. Treatments from upper left to lower right: complete, control, no biotin, no amino acids, no magnesium sulfate, no thiamine, no spermidine, and a plate with a co-culture of host and parasite for comparison. A limited amount of axenic growth was observed on the complete and the complete without biotin treatments after one week of growth. Experiments were performed in duplicate.

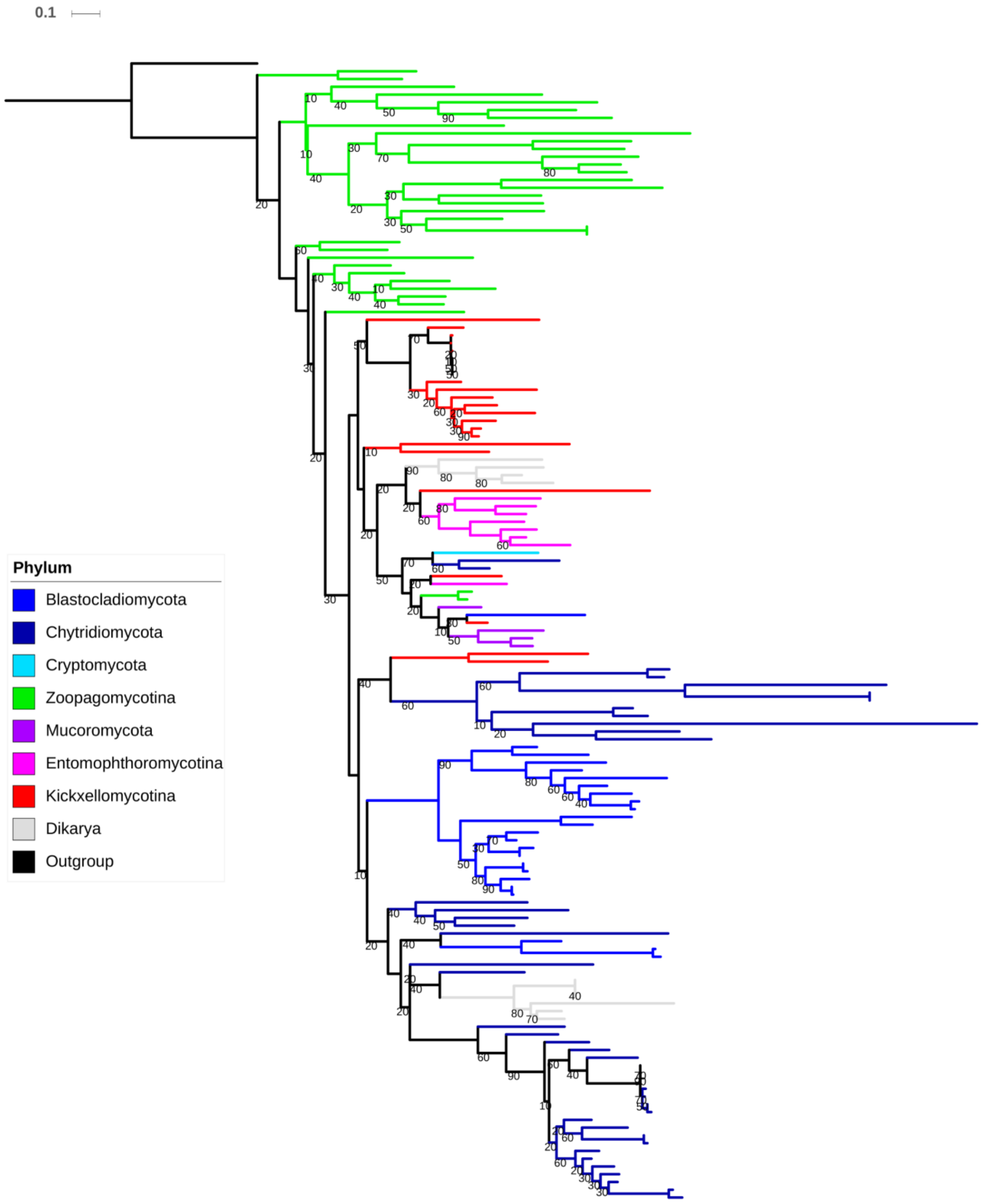

Supplementary Figure 5. Metallopeptidases were identified in early-diverging lineages, with a specific group (green) derived from mycoparasitic species of Zoopagomycotina.

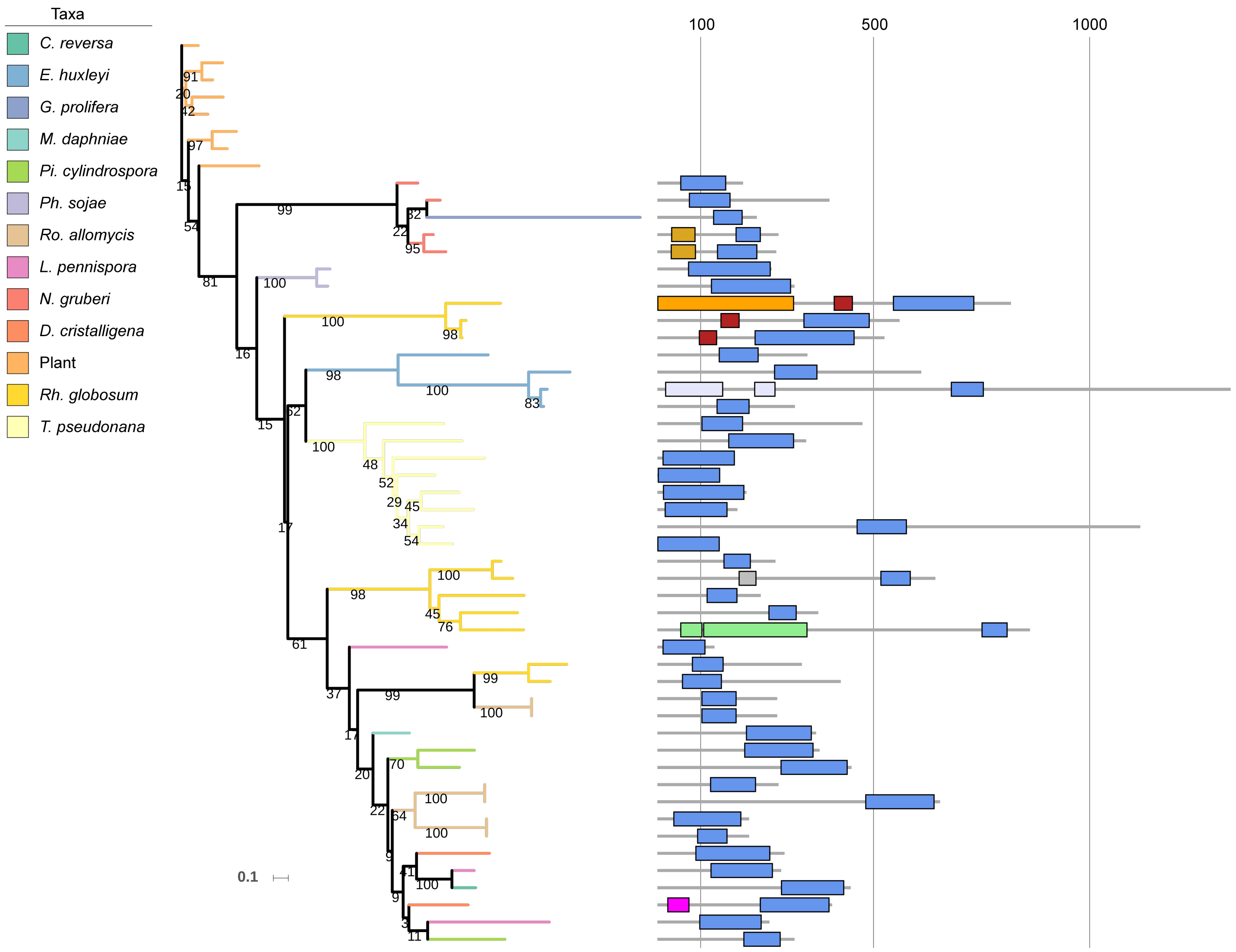

Supplementary Figure 6. Chitinases of the Glycoside Hydrolase 19 (GH19) family were identified in early-diverging fungal lineages, including among the Chytridiomycota and Zoopagomycota. A gene tree illustrates their relationship to plant and other non-fungal Eukaryote chitinases, and a domain schematic illustrates placement of functional domains along the protein. Blue = GH19 chitinase (PF00182]; Dark orange = SH3 domain [PF08239]; Light orange = GH18 chitinase [PF00704]; Red = Carbohydrate binding module (CBM) 5/12 [PF02839]; Lavender = LPMO 10 [PF03067]; Green = β–lactamase [PF00144]; Grey = LysM [PF01476]; Pink = CBM 19 [PF03427]

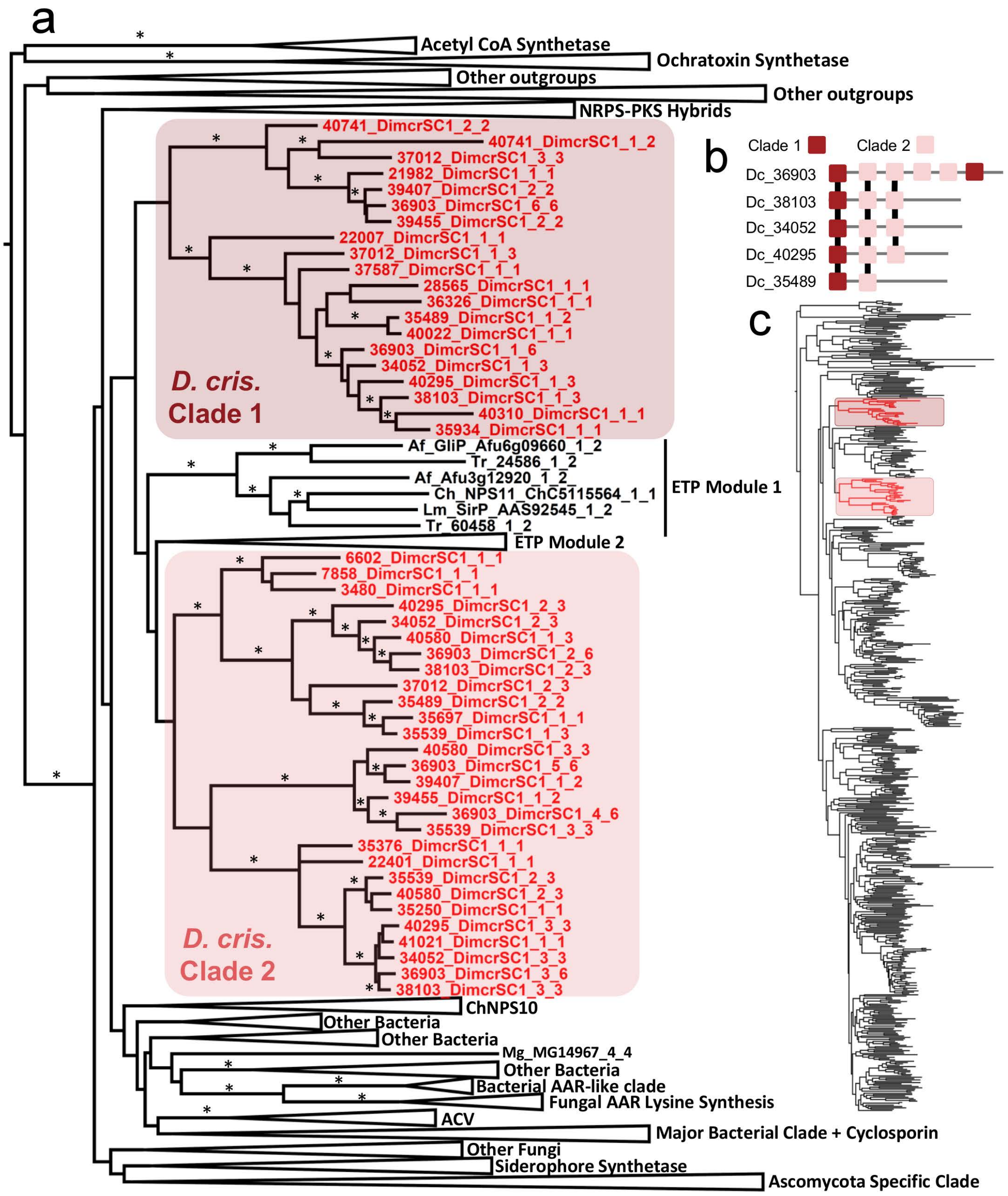

Supplementary Figure 7. Secondary metabolite expansion in *D. cristalligena*. a) Phylogenetic reconstruction of the Nonribosomal Peptide Synthetase (NRPS) adenylation domains from across fungi and the newly sequenced, early-diverging fungal genomes, highlighting the two lineage specific expansions in *D. cristalligena* (Clades 1 and 2). b) Five *D. cristalligena* NRPS genes have multiple adenylation domains per gene. Phylogenetic relationships between adenylation domains of these five proteins is represented by thick black bars (i.e. the first adenylation domain of all five proteins are homologous, as are the second and third). c) Skeleton phylogeny showing the full breadth of full adenylation domain sampling and placement of highlighted clades.

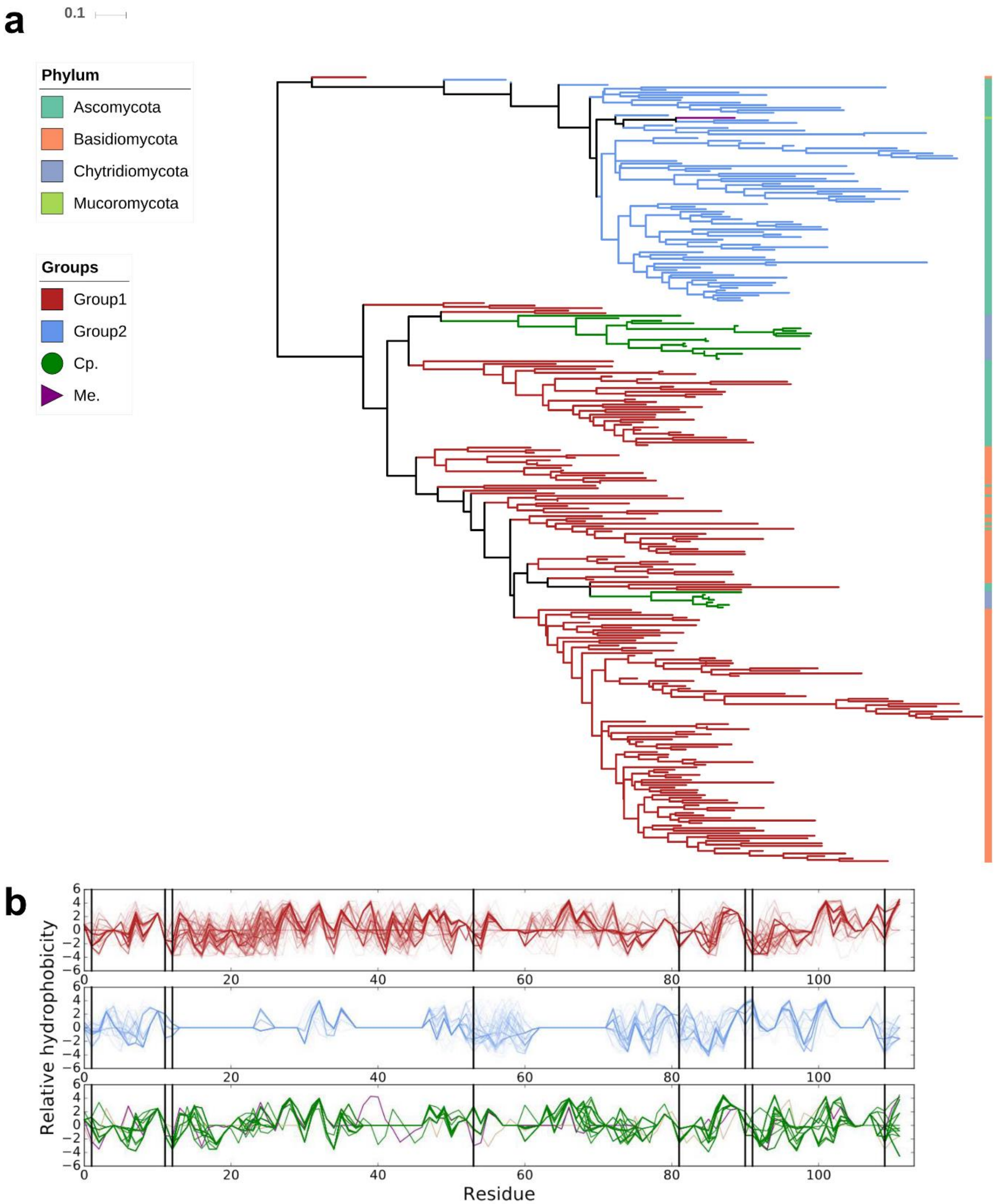

Supplementary Figure 8. Fungal Hydrophobins identified outside of the Dikarya. a) RAxML tree of hydrophobins taken from PFAM seed set (Group 1: PF01185; Group 2: PF06766), and those identified in *C. protostelioides*, and *M. elongata*. Tree branches are colored according to Group (Red, Group 1; Blue, Group 2) where known and species (Green, *C. protostelioides*; Purple, *M. elongata*) where unknown. b) Hydropathy plots generated from hydrophobin sequences. Black vertical lines indicate conserved cysteine residues.

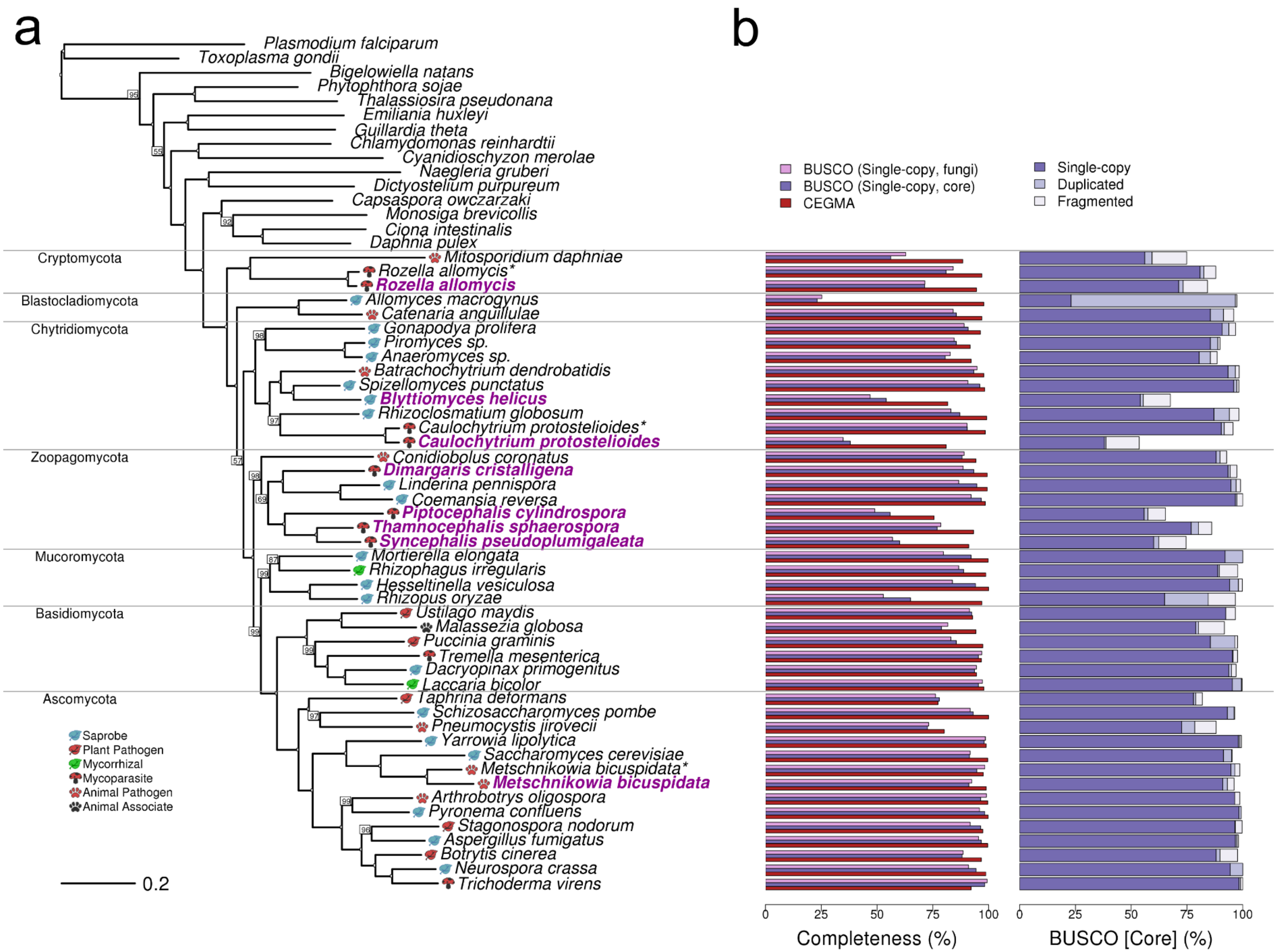

Supplementary Figure 9. Comparison of BUSCO and CEGMA. a) RAxML tree constructed from MCL clustering from across the Fungi and deep branching eukaryote outgroups. Support values based on 1000 bootstrap replicates. Bootstrap values (< 100%) are indicated on branches. Fungal species are annotated with simplified lifestyle icons. Species sequenced using single-cell methods are colored in magenta. Enrichment cultures for *R. allomycis* and *C. protostelioides*, and a related species of *M. bicuspidata* are denoted with an asterisk. b) Comparision of completeness methods CEGMA and two BUSCO datasets (fungal and core eukaryotic), and details (single-copy, fragmentation, and duplication) of the completeness assessment using the BUSCO core dataset.

Supplementary Datasets

Description: Nexus formatted files, each of which contains one or more RAxML phylogenetic trees. Files were used for topology comparison of single- or multi-cell libraries relative to the tree built with co-assemblies.

Contents:

1. Caupr_multitree.nex
   - 21 KB Nexus file
   - Contains 6 phylogenetic trees, each using a different library of *Caulochytrium protostelioides*: one 1-cell, one 10-cell, and four 100-cell libraries
2. Dimcr_multitree.nex
   - 45 KB Nexus file
   - Contains 14 phylogenetic trees, each using a different library of *Dimargaris cristalligena*: eight 1-cell, one 50-cell, and five 100-cell libraries
3. Metbi_multitree.nex
   - 18 KB Nexus file
   - Contains 5 phylogenetic trees, each using a different library of *Metschnikowia bicuspidata*: three 1-cell, one 10-cell, and one 100-cell libraries
4. Rozal_multitree.nex
   - 44 KB Nexus file
   - Contains 14 phylogenetic trees, each using a different library of *Rozella allomycis*: eleven 1-cell and three 100-cell libraries
5. Fig1_tree.nex
   - 5 KB Nexus file
   - Contains 1 phylogenetic tree, built using the co-assemblies and presented graphically as Figure 1
