# Supplementary material for: Leveraging single-cell genomics to expand the fungal tree of life
Source: Nat Microbiol. 2018 Oct 8;3(12):1417–28. doi: 10.1038/s41564-018-0261-0 (PMC6784888; doi:10.1038/s41564-018-0261-0)
Supplement: Supplementary file 2 — Reporting Summary [file 41564_2018_261_MOESM2_ESM.pdf]

nature research

Corresponding author(s): Igor V. Grigoriev
Timothy Y. James

Reporting Summary

Nature Research wishes to improve the reproducibility of the work that we publish. This form provides structure for consistency and transparency in reporting. For further information on Nature Research policies, see Authors & Referees and the Editorial Policy Checklist.

Statistical parameters

When statistical analyses are reported, confirm that the following items are present in the relevant location (e.g. figure legend, table legend, main text, or Methods section).

n/a	Confirmed	
☐	☒	The exact sample size (n) for each experimental group/condition, given as a discrete number and unit of measurement
☐	☒	An indication of whether measurements were taken from distinct samples or whether the same sample was measured repeatedly
☒	☐	The statistical test(s) used AND whether they are one- or two-sided<br>*Only common tests should be described solely by name; describe more complex techniques in the Methods section.*
☒	☐	A description of all covariates tested
☒	☐	A description of any assumptions or corrections, such as tests of normality and adjustment for multiple comparisons
☐	☒	A full description of the statistics including central tendency (e.g. means) or other basic estimates (e.g. regression coefficient) AND variation (e.g. standard deviation) or associated estimates of uncertainty (e.g. confidence intervals)
☒	☐	For null hypothesis testing, the test statistic (e.g. F, t, r) with confidence intervals, effect sizes, degrees of freedom and P value noted<br>*Give P values as exact values whenever suitable.*
☒	☐	For Bayesian analysis, information on the choice of priors and Markov chain Monte Carlo settings
☒	☐	For hierarchical and complex designs, identification of the appropriate level for tests and full reporting of outcomes
☒	☐	Estimates of effect sizes (e.g. Cohen's d, Pearson's r), indicating how they were calculated
☒	☐	Clearly defined error bars<br>*State explicitly what error bars represent (e.g. SD, SE, CI)*

Our web collection on statistics for biologists may be useful.

Software and code

Policy information about availability of computer code

Data collection	SPAdes (Bankevich et al. 2012)<br>JGI Annotation Pipeline (Grigoriev et al. 2014)

Data analysis	CLANS (Frickey & Lupas 2004)<br>BWA (Li 2009)<br>FreeBayes (Garrison & Marth 2012)<br>BBTools (http://jgi.doe.gov/data-and-tools/bbtools/)<br>RAxML (Stamatakis 2014)<br>Muscle (Edgar 2004)<br>HMMER (Eddy 2011)<br>MAFFT (Katoh & Standley 2013)<br>MCL (Enright, Van Dongen, & Ouzonis, 2012)<br>PRIAM (Claudel-Renard et al. 2003)<br>APE [R package] (Paradis et al. 2004)<br><br>IPath (Yamada et al. 2011)<br>IToL (Letunic & Bork 2016)

For manuscripts utilizing custom algorithms or software that are central to the research but not yet described in published literature, software must be made available to editors/reviewers upon request. We strongly encourage code deposition in a community repository (e.g. GitHub). See the Nature Research guidelines for submitting code & software for further information.

Data

Policy information about availability of data

All manuscripts must include a data availability statement. This statement should provide the following information, where applicable:

- Accession codes, unique identifiers, or web links for publicly available datasets
- A list of figures that have associated raw data
- A description of any restrictions on data availability

The co-assembled genomes and annotations of the target species are available through MycoCosm (https://genome.jgi.doe.gov/fungi) and Genbank using the following MycoCosm URLs and NCBI accessions, respectively: Rozella allomycis CSF55 single-cell (https://genome.jgi.doe.gov/Rozal_SC1; QUVT00000000), B. helicus Perch Fen single-cell (https://genome.jgi.doe.gov/Blyhe1; QPFV00000000), Caulochytrium protostelioides ATCC 52028 single-cell (https://genome.jgi.doe.gov/Caupr_SCcomb; QUVS00000000), Dimargaris cristalligena RSA 468 single-cell (https://genome.jgi.doe.gov/DimcrSC1; QRFA00000000), Piptocephalis cylindrospora RSA 2659 single-cell (https://genome.jgi.doe.gov/Pipcy3_1; QPFT00000000), Thamnocephalis sphaerospora RSA 1356 single-cell (https://genome.jgi.doe.gov/Thasp1; QUVU00000000), Syncephalis pseudoplumigaleata Benny S71-1 single-cell (https://genome.jgi.doe.gov/Synps1; QUVV00000000), and Metschnikowia bicuspidata single-cell (https://genome.jgi.doe.gov/Metbi_SCcomb; QUVR00000000). The whole genome sequence for the non-single-cell isolate C. protostelioides ATCC 52028 is available through MycoCosm (https://genome.jgi.doe.gov/Caupr1) and Genbank (QAJV00000000). The whole genome sequences for the non-single-cell isolate of R. allomycis CSF55 was not determined in this study, and is available through MycoCosm (https://genome.jgi.doe.gov/Rozal1_1) and Genbank (ATJD00000000). The genome sequence for the non-single-cell M. bicuspidata NRRL YB-4993 was also not determined in this study, and is available through MycoCosm (https://genome.jgi.doe.gov/Metbi1) and Genbank (LXTC00000000).

Field-specific reporting

Please select the best fit for your research. If you are not sure, read the appropriate sections before making your selection.

☒ Life sciences ☐ Behavioural & social sciences ☐ Ecological, evolutionary & environmental sciences

For a reference copy of the document with all sections, see nature.com/authors/policies/ReportingSummary-flat.pdf

Life sciences study design

All studies must disclose on these points even when the disclosure is negative.

Sample size	The fungal and eukaryotic taxa used for comparative analyses were selected to best represent the breadth of fungal diversity. In general, sample sizes represent the extent of available data.
Data exclusions	Exclusions were limited to institutional standard quality control filtering of sequencing data.
Replication	For single-cell sequencing, multiple genome libraries were constructed for each species considered. Multiple individual libraries were constructed from singular individual cells. Additionally, multiple individual libraries were also constructed from pools of individual cells. Cells from a given species were isolated from the same culture.
Randomization	Group allocation was not a part of this study, therefore there was no randomization.
Blinding	Group allocation was not a part of this study, therefore there was no blinding.

Reporting for specific materials, systems and methods

Materials & experimental systems

n/a	Involved in the study
☒	☐ Unique biological materials
☒	☐ Antibodies
☒	☐ Eukaryotic cell lines
☒	☐ Palaeontology
☒	☐ Animals and other organisms
☒	☐ Human research participants

Methods

n/a	Involved in the study
☒	☐ ChIP-seq
☒	☐ Flow cytometry
☒	☐ MRI-based neuroimaging
